# Boosting Electrochemical Urea Synthesis via Constructing Ordered Pd–Zn Active Pair

**DOI:** 10.1007/s40820-024-01462-w

**Published:** 2024-07-15

**Authors:** Weiliang Zhou, Chao Feng, Xuan Li, Xingxing Jiang, Lingyan Jing, Shuai Qi, Qihua Huo, Miaoyuan Lv, Xinbao Chen, Tianchi Huang, Jingwen Zhao, Na Meng, Hengpan Yang, Qi Hu, Chuanxin He

**Affiliations:** 1 College of Chemistry and Environmental Engineering, Shenzhen University, Shenzhen, 518060 Guangdong People’s Republic of China; 2 School of Chemistry and Chemical Engineering/State Key Laboratory Incubation Base for Green Processing of Chemical Engineering, Shihezi University, Shihezi, 832003 Xinjiang People’s Republic of China

**Keywords:** Electrochemical C–N coupling, Urea electrosynthesis, Intermetallic compounds, Geometric structures, Active pairs

## Abstract

Electrochemical co-reduction of nitrate (NO_3_^–^) and carbon dioxide (CO_2_) has been widely regarded as a promising route to produce urea under ambient conditions, however the yield rate of urea has remained limited. Here, we report an atomically ordered intermetallic pallium-zinc (PdZn) electrocatalyst comprising a high density of PdZn pairs for boosting urea electrosynthesis. It is found that Pd and Zn are responsible for the adsorption and activation of NO_3_^–^ and CO_2_, respectively, and thus the co-adsorption and co-activation NO_3_^–^ and CO_2_ are achieved in ordered PdZn pairs. More importantly, the ordered and well-defined PdZn pairs provide a dual-site geometric structure conducive to the key C–N coupling with a low kinetical barrier, as demonstrated on both operando measurements and theoretical calculations. Consequently, the PdZn electrocatalyst displays excellent performance for the co-reduction to generate urea with a maximum urea Faradaic efficiency of 62.78% and a urea yield rate of 1274.42 μg mg^–1^ h^–1^, and the latter is 1.5-fold larger than disordered pairs in PdZn alloys. This work paves new pathways to boost urea electrosynthesis via constructing ordered dual-metal pairs.

**Supplementary Information:**

The online version contains supplementary material available at 10.1007/s40820-024-01462-w.

## Highlights

- Ordered intermetallic pallium-zinc (PdZn) electrocatalyst comprising a high density of PdZn pairs is synthesized for achieving co-adsorption and co-activation carbon dioxide and nitrate.
- Both operando measurements and theoretical calculations reveal that the PdZn pairs provide a dual-site geometric structure conducive to the key C–N coupling with a low kinetical barrier.
- The intermetallic PdZn displays excellent performance for the co-reduction of nitrate (NO_3_^–^) and carbon dioxide (CO_2_) toward urea, with a maximum FE_urea_ of 62.78% at a small potential of – 0.4 V versus RHE.

**Supplementary Information:**

The online version contains supplementary material available at 10.1007/s40820-024-01462-w.

## Graphical abstract

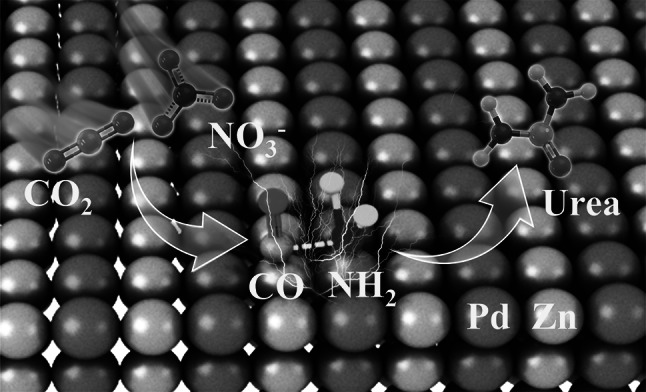

## Introduction

As an important nitrogen fertilizer, urea has demonstrated its crucial role in increasing grain yield [1–3]. Unfortunately, the mass production of urea is mainly via the coupling of carbon dioxide and liquid ammonia under harsh conditions (i.e., high temperature and pressure), which requires high energy consumption and expensive liquid ammonia as a reactant [4–6]. Therefore, it is highly desirable to develop an energy- and cost-saving route for urea production [7–10]. Currently, the electrochemical co-reduction of nitrate (NO_3_^–^) and carbon dioxide (CO_2_) has emerged as a promising route for urea synthesis due to its mild reaction conditions and relatively low energy consumption. Another benefit is that NO_3_^–^ can come from wastewater, which helps to address the environmental issue of NO_3_^–^ and thus promote nitrogen cycle [11–15].

The process of NO_3_^–^/CO_2_ co-reduction to generate urea is extremely complex involving 18 protons-coupled 16 electrons transfer. Consequently, a variety of C and N byproducts (i.e., CO, C_2_H_4_, NO_2_^–^, and NH_3_) are always simultaneously generated during the co-reduction along with unavoidable H_2_ from the competitive hydrogen evolution reaction (HER), leading to unsatisfied Faradaic efficiency (FE_urea_) of urea [16–24]. To this end, great efforts have been devoted to develop efficient and selective electrocatalysts for boosting urea generation from the co-reduction [25–28]. Considering that the co-reduction comprises two subreactions of the CO_2_ reduction reaction (CO_2_RR) and NO_3_^–^ reduction reaction (NO_3_^–^RR), a qualified co-reduction electrocatalyst should meet at least following three requirements: good co-adsorption and co-activation ability for both CO_2_ and NO_3_^–^, geometric locations of active sites conducive to the key C–N coupling, and strong ability for suppressing by-products generation [29–31]. Since a single active site prefers dsorption and activation of specific C or N reactants, designing electrocatalysts with a single active site that meet all above three requirements remain a huge challenge. To break the adsorption limitation of single active site, adjacent Fe–Ni diatomic pairs, in which Fe sites for the NO_3_^–^ reduction and Ni sites for CO_2_ reduction, were constructed to boost the formation of C/N intermediates and subsequent C–N coupling, thus achieving a significantly increased FE_urea_ than the counterpart with a single Fe or Ni site [30]. Therefore, to promote urea generation from the co-reduction of CO_2_/NO_3_^–^, rationally designing and constructing efficient dual active pairs are highly desirable.

Recently, the construction of diatomic pairs comprising two nearby single atomic metals has emerged as a robust strategy to boost various electrocatalytic reactions due to the powerful synergetic effect between two atomic sites [30, 38–40]. However, the precise synthesis of such diatomic sites with a high density is really difficult, which greatly limits the selectivity and activity of these electrocatalysts. We note that intermetallic compounds naturally comprise two different kinds of metal atoms that are orderly arranged in alternating rows, thereby offering a high density of dual-metal pairs [41–43]. Moreover, in such metal pairs, the distance between two metal compositions is definite, which provides a fully consistent dual-metal geometrical structure to avoid by-product generation resulted from inconsistent active sites. Therefore, we conceived that creating intermetallic compounds with ordered and efficient metal pairs may significantly promote urea electrosynthesis while suppress by-products generation, however related research on this subject is still limited.

Herein, we design a PdZn intermetallic electrocatalyst containing a high density of ordered PdZn pairs for boosting the urea electrosynthesis. Structural characterizations indicate that Pd and Zn atoms are orderly arranged in alternatingly rows, which makes Pd and Zn atoms closely connected. By systemically investigating the performance of pure Pd, pure Zn, and intermetallic PdZn for subreactions of NO_3_^–^RR and CO_2_RR, it is observed that Pd and Zn are active for NO_3_^–^RR and CO_2_RR, respectively. Accordingly, in ordered PdZn pairs, the co-adsorption and co-activation of NO_3_^–^ and CO_2_ are achieved to generate numerous *NH_2_ and *CO intermediates. Both density functional theory (DFT) calculations and operando infrared spectra reveal the presence of strongly electronic coupling effect between nearby Pd and Zn atoms in ordered PdZn pairs for reducing the energy barrier of NO_3_^−^RR and CO_2_RR, and the geometric structure of dual-metal sites to C−N bonds can smoothly drive the key C–N coupling with a small kinetic barrier. Consequently, the intermetallic PdZn enables a high urea yield rate at a small potential of – 0.4 V versus reversible hydrogen electrode (RHE), significantly outperforming disordered PdZn alloys and most of other reported electrocatalysts.

## Experimental Section

### Material Syntheses

#### Synthesis of PdZn/C

The ordered PdZn intermetallic compounds were synthesized according to previous literatures with slight modifications [42]. In a typical process, 0.2 mmol PdCl_2_ and 0.2 mmol ZnCl_2_ were dissolved in 20 mL deionized water to generate a clear solution at 60 °C. Afterward, 77 mg porous carbon (Vulcan XC-72) was added into the solution under stirring, and then the water was completely evaporated at 60 °C for 4 ~ 5 h, followed by vacuum drying for 6 h to obtain black powders. Finally, the black powders were calcinated at 300 °C in H_2_ for 2 h to obtain disordered PdZn alloys (denoted D-PdZn/C). As for the ordered PdZn intermetallic compounds, the calcination temperature in H_2_ increased to 500 °C, and the resulting product was denoted O-PdZn/C.

#### Synthesis of Pd/C

The synthesis process of Pd/C was similar as that of D-PdZn/C, except that without Zn element was added and calcination conditions changed to 150 °C in H_2_ for 2 h.

#### Synthesis of Zn/C

The Zn/C sample was prepared via the electroreduction of ZnO/C. Firstly, ZnO/C was synthesized through the similar process as that of D-PdZn/C, except that without Pd element was added and calcination conditions changed to 500 °C in Ar for 2 h. Afterward, the ZnO/C was loaded on a carbon cloth substrate, and ZnO was then reduced to Zn through the electrochemical reduction at – 2.1 V versus RHE in an Ar-saturated 0.2 M KHCO_3_ solution for 30 min. The obtained product was denoted Zn/C.

### Material Characterization

Morphologies of different materials were investigated by field emission scanning electron microscopy (FE-SEM) on a JEOL JSM-7800F. Images of transmission electron microscopy (TEM), high-angle annular dark-field scanning transmission electron microscopy (HAADF-STEM), and elemental mapping were collected on a JEOL JEM-F200. 2020 sorptometer. X-ray diffraction (XRD) patterns were recorded on a D8 ADVANCE diffractometer with Cu Kα radiation. The chemical state of different materials was investigated by using a Thermo VG Scientific ESCALAB 250 X-ray photoelectron spectrometer (Thermo Electron, U.K.).

### Electrochemical Measurements

Electrochemical measurements were carried out using a CHI760E electrochemical workstation and a three-electrode system, which included a piece of carbon paper (1 × 1 cm^2^) loaded with electrocatalysts, an Ag/AgCl electrode, and a piece of Pt foil as the working electrode, reference electrode and counter electrode, respectively. The cathode and anode were separated by a piece of Nafion@117 cation exchange membrane in a typical H-Cell, and the electrolyte was CO_2_-saturated 0.1 M KNO_3_ and 0.2 M KHCO_3_. The catalyst ink was prepared by dispersing 2 mg electrocatalyst in a mixed solution of 950 μL ethanol and 50 μL Nafion (5 wt% aqueous solution) under sonication for 30 min, and then 100 μL inks were loaded on carbon paper as the working electrode. Besides, we investigated the influence of catalyst mass loading on the performance of urea electrosynthesis, signifying that the optimal loading is 0.2 mg cm^−2^ (Fig. S1). All LSV curves were collected without IR compensation, and all the potentials were converted into the reversible hydrogen electrode (RHE) scale using the following equation:${\text{E}}_{{{\text{RHE}}}} \, = \,{\text{E}}_{{\text{Ag/AgCl}}} \, + \,0.2046\, + \,0.0591\, \times \,{\text{pH}}.$

## Results and Discussion

### Structural Characterization

**Fig. 1:**
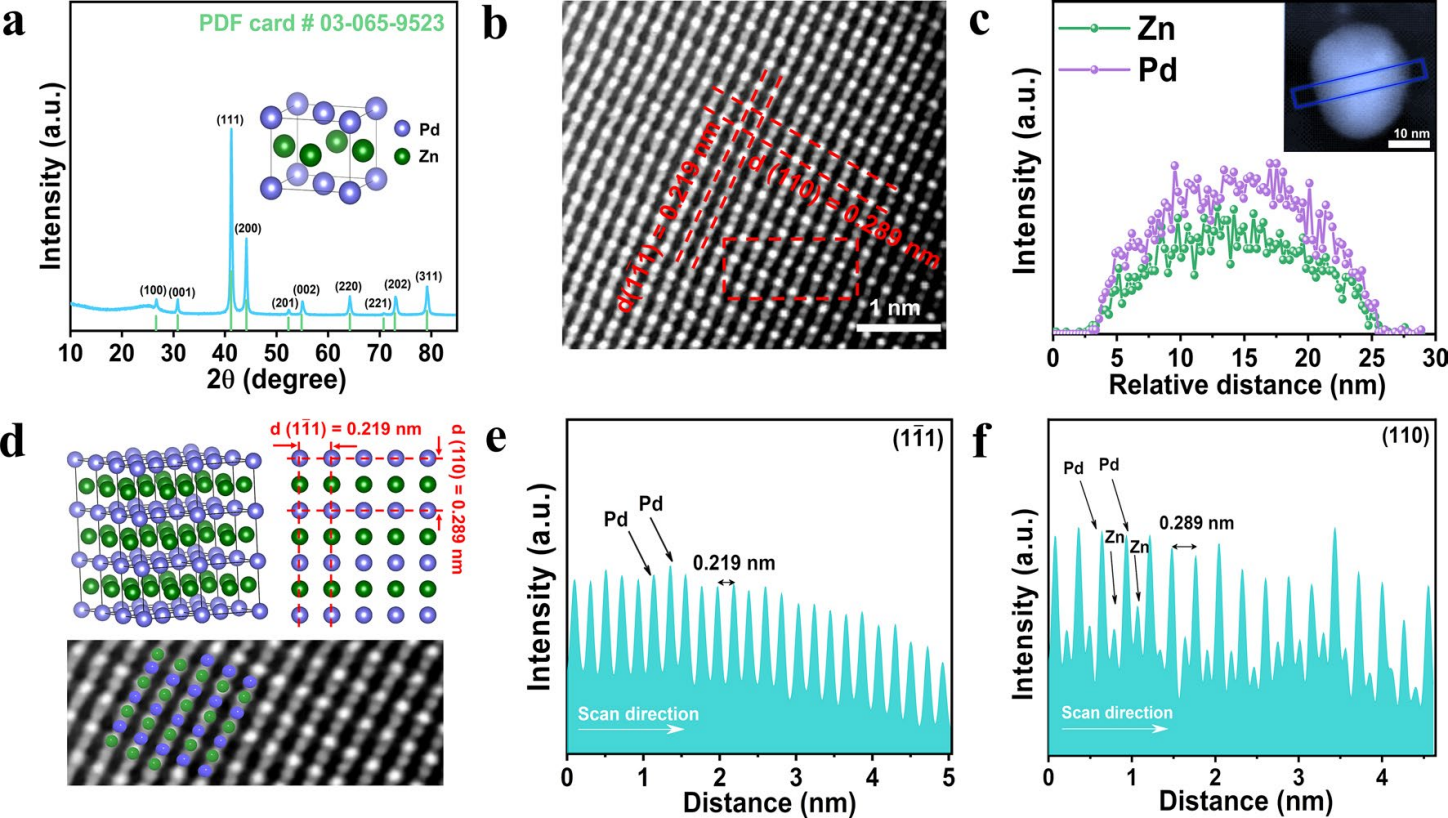
Structural characterization. **a** XRD pattern of O-PdZn/C. **b** Aberration-corrected HAADF–STEM images of O-PdZn/C. **c** EELS line scan profile extracted from the dashed box in the inset. **d** Three-dimensional crystal structure diagram and two-dimensional planar diagram of O-PdZn/C, and the enlarged STEM images of **b**. **e, f** Intensity profiles measured from HAADF–STEM images of O-PdZn/C form the crystal planes of (1$\bar{1}{1}$) and (110)

Considering that metallic Pd and Zn are highly active for the NO_3_^–^RR and CO_2_RR [44–48], respectively, we selected Pd and Zn to construct an ordered intermetallic PdZn compound (O-PdZn) comprising a high density of Pd-Zn pairs, aiming to boost the generation of both C and N intermediates and subsequent coupling of these intermediates to generate urea. O-PdZn was synthesized via a traditional impregnation method including the first step of metal ion impregnation on porous carbon supports and subsequent high-temperature reduction in H_2_ atmosphere, and the resulting sample was denoted as O-PdZn/C. Figure 1a showed the X-ray diffraction (XRD) pattern of O-PdZn/C, which was well indexed to an ordered tetragonal structure (JCPDS: 03-065-9523). Since the crystal structure of O-PdZn/C was different from that of Pd/C and Zn/C, the diffraction peaks of Pd/C and Zn/C disappeared and the new diffraction peaks appeared in the O-PdZn/C [49]. For comparison, pure Pd, pure Zn, and disordered PdZn alloy nanoparticles were also synthesized on the porous carbon as control samples (Detailed synthetic processes see Experimental section), which were denoted as Pd/C, Zn/C, and D-PdZn/C, respectively, and corresponding XRD patterns were shown in Figs. S2 and S3. Moreover, owing to the lattice contraction caused by the entry of Zn atoms into the lattice matrix of Pd atoms, the diffraction peaks of Zn element disappeared, and the diffraction peaks at 40.46° and 47.02° for metallic Pd on D-PdZn/C shifted to higher diffraction angles compared to Pd/C. Transmission electron microscopy (TEM) images of O-PdZn/C displayed the nanoparticles uniformly dispersed on the porous carbon with the average diameter of 20.98 $\pm$ 0.1 nm (Fig. S4), and corresponding energy-dispersive X-ray spectroscopy (EDX) elemental mappings confirmed that Pd and Zn elements shared similar distributions (Figs. S5, S6). As determined by Inductively Coupled Plasma Optical Emission Spectrometer (ICP-OES), the atomic ratio of Pd/Zn was ~ 1 (Table S1), further confirming the generation of PdZn intermetallic. TEM images and average diameter of control samples were shown in Figs. S7-S9, and the results indicated that the size of O-PdZn/C (21 nm) was larger than that of D-PdZn/C (15 nm) due to the higher annealing temperatures in O-PdZn/C. Moreover, the lattice spacing of D-PdZn/C was 0.211 nm corresponding to the (111) plane of disordered PdZn alloys. X-ray photoelectron spectroscopy (XPS) measurements were performed to gain information about the chemical compositions and valence states. As depicted in Fig. S10, the XPS survey spectrum of O-PdZn/C revealed the presence of Pd, Zn, O, and C elements. Moreover, binding energies of Pd 3*d* for O-PdZn/C shifted to lower values compared to pure Pd, while binding energies of Zn 2*p* for O-PdZn/C shifted to higher values with respect to pure Zn, suggesting the existence of strong electronic coupling effect between Pd and Zn. The atomic arrangement of Pd and Zn in O-PdZn was further examined by using aberration-corrected high-angle annular dark-field scanning transmission electron microscopy (HAADF-STEM). The entire lattice displayed as fully ordered rectangular arrays containing alternating bright and dark spots columns corresponding to Pd and Zn atoms, respectively (Fig. 1b). Moreover, the lattice spacing of 0.219 and 0.289 nm were well accordance with (110) and (1$\bar{1}{1}$) planes of O-PdZn. EDX elemental line profiles, extracted from dashed boxes in insert of Fig. 1c, showed that Pd and Zn shared quite similar bell-shaped line profiles (Fig. 1c), suggesting that Pd and Zn elements were well mixed and uniformly distributed through the entire particle. The 3D model of the unit cell and the corresponding 2D planar graph of O-PdZn/C were then plotted in Fig. 1d, and two vertically oriented unit cell are highlighted and marked in the (110) and (1$\bar{1}{1}$) directions. Moreover, the intensity profiles obtained from STEM images in both (110) and (1$\bar{1}{1}$) directions manifested a similar periodic oscillation pattern (Fig. 1e, f), further confirming the formation of atomic ordered PdZn pairs in O-PdZn/C sample.

### Electrochemical Performance

**Fig. 2:**
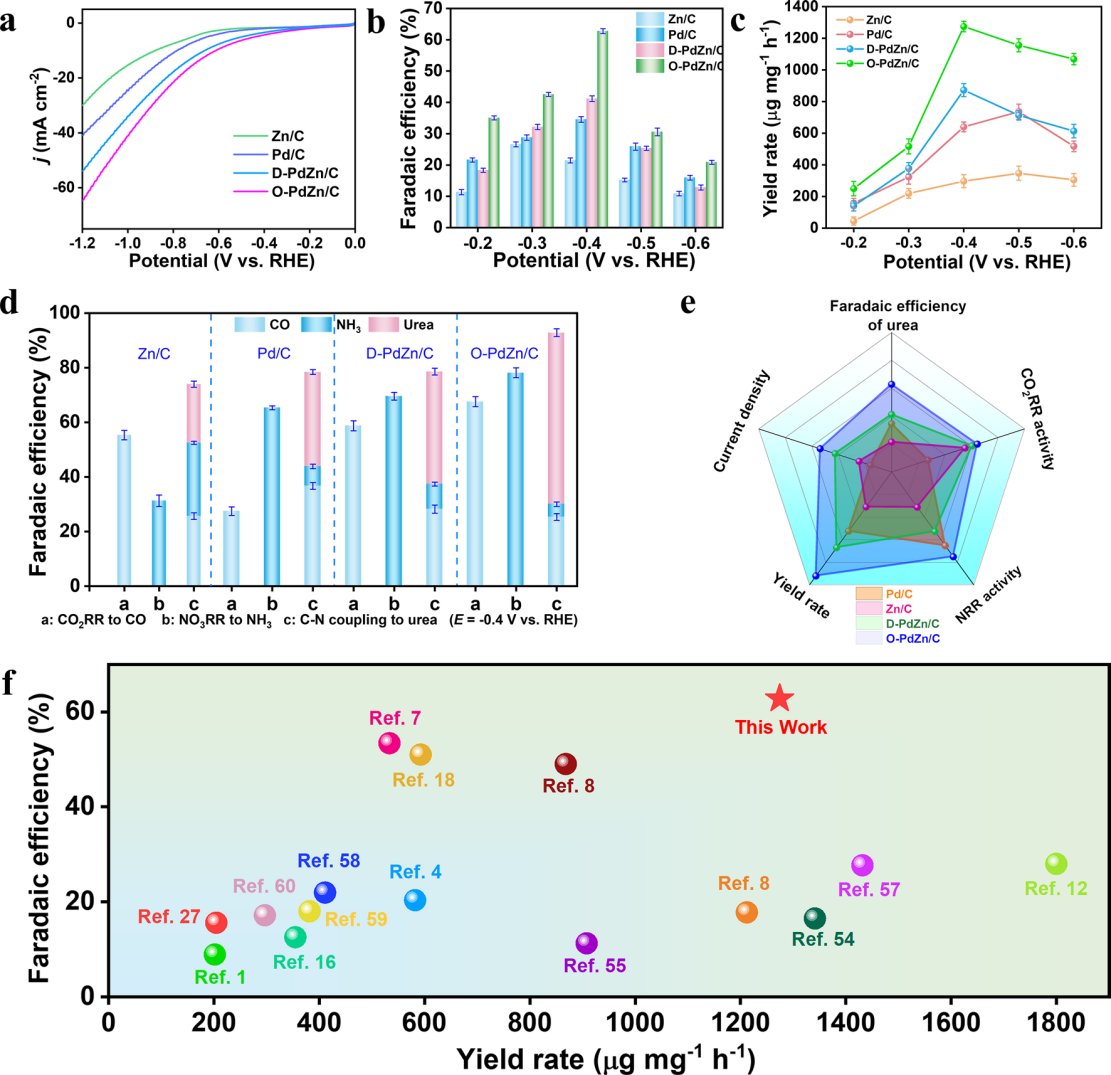
Performance characterization. **a** LSV curves of co-reduction for various catalysts. **b** Faradaic efficiencies of urea. **c** Yield rate of urea on various catalysts at various potentials. **d** The product distributions of CO_2_RR, NO_3_^−^RR, and urea synthesis on various catalysts at − 0.4 V versus RHE. **e** Illustrated correlation between CO_2_RR activity, NO_3_^−^RR activity, current density, the faradaic efficiencies and yield rates of urea over various catalysts. **f** Comparison of this work with currently reported electrocatalysts

The electrocatalytic performance of various samples for the NO_3_^–^RR, CO_2_RR, and co-reduction of NO_3_^–^/CO_2_ was evaluated in 0.1 M KNO_3_ and 0.2 M KHCO_3_ electrolyte with a typic three-electrode system. Besides, we also investigated the influence of alkali metal cations on the performance of the co-reduction reaction. Compared with the electrolyte containing Na^+^, slight larger current densities were displayed in the electrolyte containing K^+^ (Fig. S11). As it reported, the larger current densities on K^+^ can be attributed to the reduced energy barrier of C–N coupling [50]. Corresponding linear sweep voltammetry (LSV) curves of O-PdZn/C displayed larger current densities for the co-reduction compared to the single NO_3_^–^RR and CO_2_RR (Fig. S12), indicating that O-PdZn/C is indeed active for the co-reduction. And then, we compared the co-reduction activity of O-PdZn/C with other control samples (i.e., Pd/C, Zn/C and D-PdZn/C). Obviously, O-PdZn/C had superior co-reduction activity with higher current densities than other samples at the whole potential windows (Fig. 2a). To further demonstrated the superior activity of O-PdZn/C, we determined FE_urea_ values at different potentials using high-performance liquid chromatography (HPLC) (Fig. S13). As expected, O-PdZn/C had higher FE_urea_ than other samples at whole tested potential range, further confirming the superior activity for the co-reduction to generate urea. For example, at the potential of – 0.4 V versus RHE, O-PdZn/C delivered a maximum FE_urea_ of 62.78%, larger than D-PdZn/C (41.19%), Pd/C (34.54%), and Zn/C (21.45%) (Fig. 2b). With higher current densities and FE_urea_, O-PdZn/C achieved a urea yield rate (*Y*_urea_) as high as 1274.42 μg mg^–1^ h^–1^, 1.5-fold larger than D-PdZn/C, twofold larger than Pd/C, and 4.3-fold larger than Zn/C (Fig. 2c). Moreover, O-PdZn/C exhibited a higher partial current density of urea (*j*_urea_) at the whole potential windows with respect to the control samples, further confirmed that O-PdZn/C exhibited superior performance in urea electrosynthesis (Fig. S14). To further enhance accuracy of FE_urea_ determination, we also detected the concentration of the urea in liquid products via the modified diacetyl monoxime and urease decomposition [13, 26, 51], and the result of these two methods was consistent with that obtained from HPLC (Figs. S15, S16), indicating the validity of our FE_urea_ values. To investigate the origin of the urea in liquid products from the co-reduction or other impurities, we performed isotope tracer experiments by replacing ^14^NO_3_^–^ with ^15^NO_3_^–^ as the N source, and the result confirmed that the detected urea indeed originated from the electrocatalytic co-reduction rather than other impurities (Fig. S17).

Considering the different structural feature of these samples, we deduced that the generation of dual PdZn pairs especially ordered pairs was more favorable for urea generation with respect to single Pd or Zn sites. To gain further insights into the superiority of ordered PdZn pairs for boosting urea generation, we also investigated the product distribution of O-PdZn/C for the NO_3_^–^RR and CO_2_RR at – 0.4 V versus. RHE, respectively (Fig. 2d). It was found that Zn/C had a CO Faradic efficiency (FE_CO_) of 55.32% much larger than that of Pd/C (27.49%) for the CO_2_RR, whereas the NH_3_ Faradic efficiency (FE_NH3_) of Zn/C (31.26%) was much smaller than that of Pd/C (65.32%), implying that Pd and Zn exhibited a high activity for only a specific reaction, such as the NO_3_^–^RR and CO_2_RR, respectively. Accordingly, in ordered PdZn pairs of O-PdZn/C, Pd and Zn were responsible for the NO_3_^–^RR and CO_2_RR, respectively, thereby generating a variety of N and C intermediates and consequently increasing the colliding probability of these intermediates to promote the key C–N coupling. As for D-PdZn/C with disordered PdZn pairs, both FE_CO_ and FE_NH3_ were smaller than O-PdZn/C with those of ordered PdZn pairs, indicating that ordered PdZn pairs favored both the NO_3_^–^RR and CO_2_RR to generate N and C intermediates. In general, compared with disorder alloys, the two metal compositions in ordered intermetallic compounds had a stronger electronic coupling effect for enhancing electrocatalytic activity. To this end, we compared the behaviors of electron transfer between Pd and Zn on O-PdZn/C and D-PdZn/C using XPS spectra **(**Figs. S18, S19), indicating the presence of stronger electron transfer on the former than the latter. Thus, compared with D-PdZn/C, the larger FE_CO_ and FE_NH3_ on O-PdZn/C could be attributed to the stronger electronic coupling effect between Pd and Zn for efficiently tuning the electronic structure of both Pd and Zn. Moreover, in D-PdZn/C, Pd and Zn atoms were randomly distributed, leading to generating inhomogeneous active sites for promoting the formation of C and N byproducts (Figs. S20, S21). Accordingly, D-PdZn/C displayed a small FE_urea_ of 41.19% for the co-reduction. In sharp contrast, O-PdZn/C delivered a much larger FE_urea_ of 62.78% because its PdZn pairs were atomically ordered and well defined. Therefore, atomically ordered PdZn pairs not only induced strong electronic coupling effect between Pd and Zn for boosting subreactions of the NO_3_^–^RR and CO_2_RR but also provided a geometric location with definite and adjacent PdZn dual sites conducive to the key C–N coupling. With above two advantages, the urea electrosynthesis performances of O-PdZn/C was superior than the control samples (Fig. 2e) and compared favorably with most other reported electrocatalysts (Fig. 2f).

Generally, metal nanoparticles with a smaller size and a larger surface area provided more active sites for improving electrocatalytic performance [52]. However, in our work, O-PdZn/C with a larger size displayed superior performance for the co-reduction reaction than D-PdZn/C with a smaller size, suggesting that the particle size was not a crucial factor that governs the electrocatalytic performance. Besides, to exclude the influence of different particle size on the electrocatalytic performance, we normalized the yield rate of urea by double-layer capacitance (*C*_dl_) values, and O-PdZn still displayed superior electrocatalytic performance than D-PdZn, further confirming that the particle size was not a crucial factor that governs the electrocatalytic performance (Figs. S22-S24). Long-term stability is an important parameter to evaluate whether an electrocatalyst is suitable for practical applications. After eight cycle’s electrolysis, the FE_urea_ and *Y*_urea_ of the O-PdZn/C remained nearly unchanged (Fig. S25), signifying the good stability of the O-PdZn/C, and the chronoamperometry test of the control samples were also shown in Fig. S26. The fact that O-PdZn/C nanoparticles were still uniformly dispersed on the porous carbon without obvious aggregation (Fig. S27), and the crystalline phase and chemical compositions of O-PdZn retained unchanged after eight cycles electrolysis, further indicating that the good stability of the O-PdZn/C (Figs. S28–S30).

### In-situ Spectroscopy Measurements

**Fig. 3:**
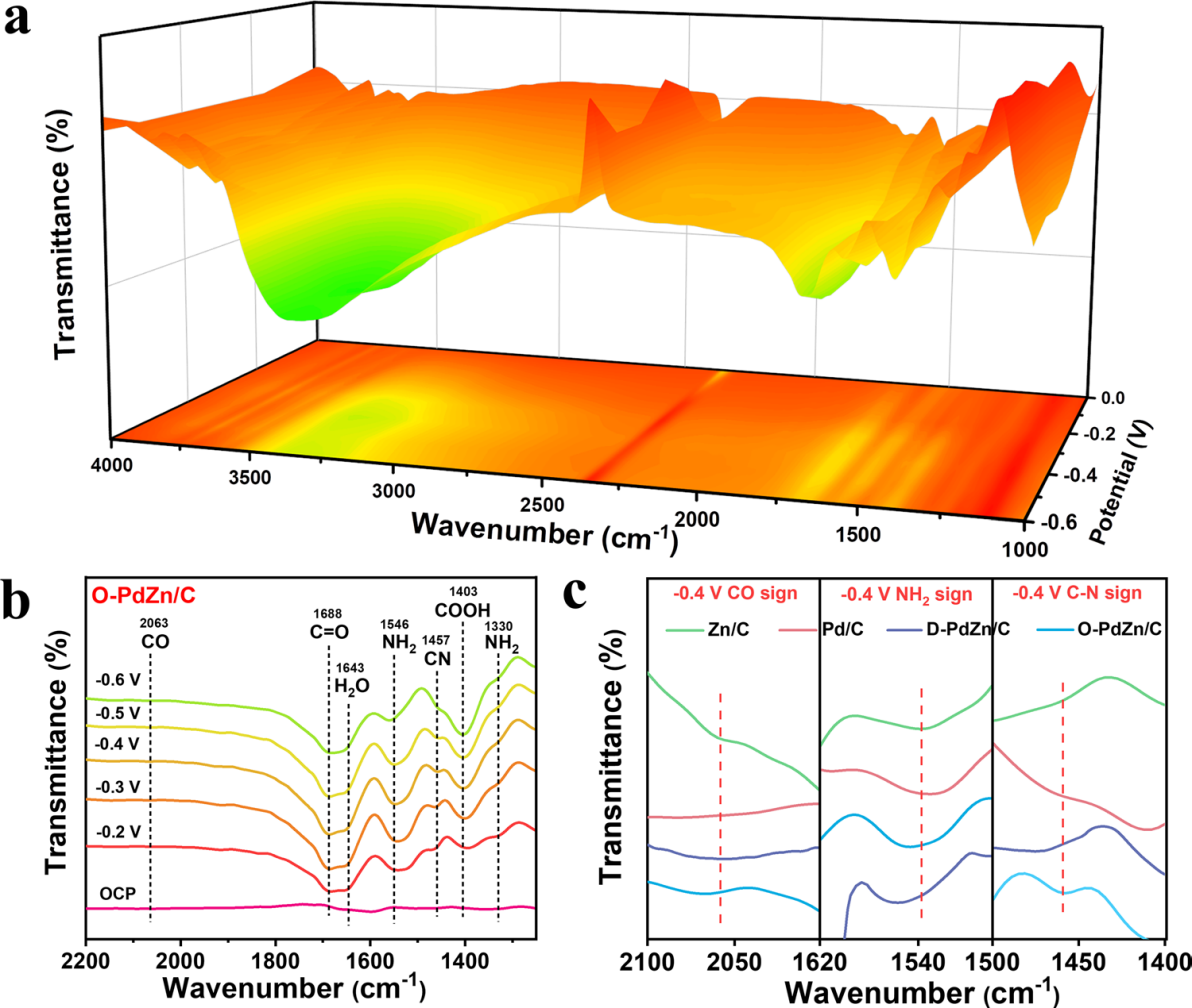
Operando ATR-FTIR spectroscopy measurements under various potentials. **a** Three-dimensional infrared signal in the range of 1000–4000 cm^−1^. **b** Infrared signals in the range of 1250–2200 cm^−1^. **c** Infrared signals of CO, NH_2_, and C–N over various catalysts at – 0.4 V versus RHE

Operando Fourier transform infrared spectroscopy (FT-IR) provides a powerful tool to investigate the evolution of reaction intermediates in real time during electrocatalytic reactions. As displayed in Fig. 3a, we recorded the IR signals at a wavenumber range from 1000 to 4000 cm^–1^ under electrocatalytic co-reduction conditions of NO_3_^–^ and CO_2_. When the applied potentials were at a range of – 0.2 to – 0.6 V versus RHE, signals of N (*NH_2_ at 1330 and 1546 cm^−1^) and C (*CO at 2063 cm^–1^, and *COOH at 1403 cm^–1^) intermediates were observed on O-PdZn/C, indicating the presence of co-activation of NO_3_^–^ and CO_2_ to generate N and C intermediates (Fig. 3b) [33, 53, 54]. An obvious IR signal at 1457 cm^–1^ for the C–N bond could also be observed, reflecting that the ordered PdZn pairs could promote the key C–N coupling process [55–60]. As it well reported, the C–N coupling from the NO_3_^–^/CO_2_ co-reduction prefers to undergo through the coupling of *NH_2_/*CO or *NH/*CO [7, 12, 29, 55]. Considering that the *NH_2_ and *CO were main N and C intermediates for the co-reduction on O-PdZn/C, we inferred that *NH_2_ and *CO were the precursors of C–N coupling over O-PdZn/C. The signal strength of *NH_2_ and *CO much stronger than that the signal of *NH on the differential electrochemical mass spectrometry (DEMS) measurement during the O-PdZn/C-catalyzed NO_3_^–^/CO_2_ co-reduction, further provided evidence that the C–N coupling was achieved by using the *NH_2_ and *CO as precursors (Fig. S31). Moreover, with respect to that on Pd/C (1654 cm^–1^), Zn/C (1660 cm^–1^) and D-PdZn/C (1651 cm^–1^), the peak of O–H on O-PdZn/C (1643 cm^–1^) red-shifted, suggesting the enhanced adsorption strength of H_2_O molecule on O-PdZn/C [61, 62], which in turn promoted the water dissociation step to provide abundant protons required by the urea electrosynthesis (Fig. S32). In addition, operando FT-IR results of Pd/C, Zn/C, and D-PdZn/C revealed that they shared the similar reaction pathway with O-PdZn/C, and the C–N coupling was also achieved by using *NH_2_ and *CO as precursors (Fig. S33). It should be noted that intensities of *NH_2_, *CO, and C–N bond on O-PdZn/C significantly enhanced with respect to that of Pd/C, Zn/C and D-PdZn (Fig. 3c), implying that the generation of ordered PdZn pairs facilitated not only subreactions of both NO_3_^–^RR and CO_2_RR for generating more N and C intermediates but also the key C–N coupling step for highly efficient urea electrosynthesis, in consistent with above electrochemical co-reduction results.

### Theoretical Insight into the Mechanism

**Fig. 4:**
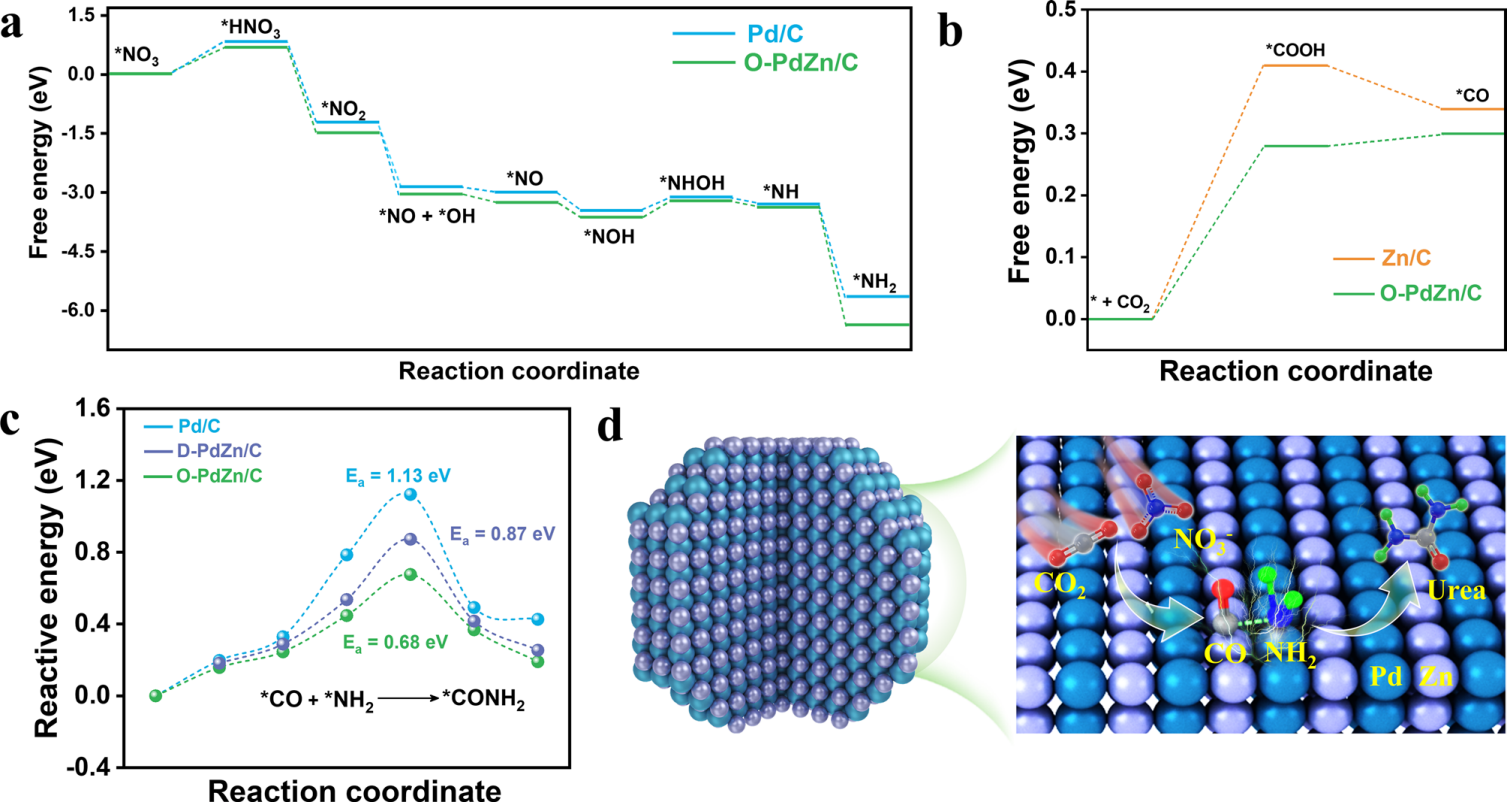
Free energy profiles. **a** *NO_3_ reduction to *NH_2_ on Pd/C and O-PdZn/C. **b** CO_2_ reduction to *CO on Zn/C and O-PdZn/C. **c** Kinetics barrier of C–N coupling for the formation of *CONH_2_. **d** Schematic representation of coupling *NH_2_ and *CO to generate urea

To further understand the enhanced mechanism of ordered PdZn pairs for urea electrosynthesis, DFT calculations were performed. We first investigated the electronic coupling effect between Pd and Zn by computing differential charge density maps (Fig. S34). The result suggested the presence of electronic transfer from Zn to Pd species, in accordance with above XPS results. Such electronic structure tuning between Zn and Pd species in PdZn pairs may alter the electrocatalytic behaviors for subreactions of the CO_2_RR and NO_3_^–^RR, respectively. To this end, we computed free energy diagrams for the electrochemical reduction of NO_3_^–^ to generate *NH_2_ intermediates on the surface of pure Pd and Pd sites of O-PdZn. As displayed in Fig. 4a, Pd sites of O-PdZn (0.71 eV) enabled a lower energy barrier of potential-determining step (PDS) for the adsorption of NO_3_^–^ than pure Pd (0.83 eV), suggesting the higher activity of Pd sites on O-PdZn for the electrochemical reduction of NO_3_^–^ to generate *NH_2_. Likewise, Zn sites of O-PdZn also exhibited a lower energy barrier and thus higher activity for the electrochemical reduction of CO_2_ to generate *CO, compared with that of pure Zn (Fig. 4b). Hence, the electronic coupling effect between Pd and Zn in O-PdZn suitably modified the electronic structure of Pd and Zn for enhancing the activity NO_3_^–^RR and CO_2_RR subreactions, respectively. Then, we computed the kinetics barrier of C–N coupling on the surface of pure Pd, D-PdZn, and O-PdZn (Fig. 4c), and the results indicated that the geometric structure of ordered PdZn pairs indeed promoted the C–N coupling, and kinetics of barrier of O-PdZn (0.68 eV) was much smaller than the pure Pd (1.12 eV) and D-PdZn (0.87 eV). Figure 4d displayed a picture that order PdZn pairs promoted the C–N coupling and urea generation. All structural models for DFT calculations were shown in Figs. S35-S39. Overall, ordered PdZn pairs in O-PdZn could not only promote key *NH_2_ and *CO intermediates generation via the strong electronic coupling effect between Pd and Zn but also provided a geometric structure conductive for the C−N coupling from *NH_2_ and *CO, thereby achieving a great improvement on yield rate of urea.

## Conclusion

In summary, we have demonstrated that an intermetallic PdZn electrocatalyst with a high density of ordered PdZn pairs could significantly promote the urea electrosynthesis from the NO_3_^–^/CO_2_ co-reduction, with a maximum *FE*_urea_ of 62.78% at a small potential of – 0.4 V versus RHE, corresponding to a *Y*_urea_ of 1274.42 μg mg^–1^ h^–1^ that displayed 1.5-fold improvement than disordered PdZn alloys and higher than most of reported electrocatalysts. Mechanism analysis, wherein we combined operando FT-IR spectra and DFT calculations, revealed that the ordered PdZn pairs not only efficiently promoted the co-adsorption and co-activation of NO_3_^–^ and CO_2_ to concurrently produce abundant *NH_2_ and *CO intermediates but also provided a dual-metal geometric structure that conducive to the key C–N coupling from *NH_2_ and *CO yet suppresses NH_3_ and CO by-products generation. It should be noted that the atomically ordered arrangement is key to the excellent electrocatalytic performance because it ensures that geometric and electronic structure of PdZn pairs are consistent throughout the entire electrocatalyst. We believed that the fresh concept of designing efficient and ordered dual-metal pairs can be extended to develop a variety of advanced electrocatalysts for enhancing activity and selectivity of other important electrochemical coupling reactions.

## Supplementary Information

Below is the link to the electronic supplementary material.

Supplementary file1 (DOC 14196 KB)
