# Supplementary material for: Boosting Electrochemical Urea Synthesis via Constructing Ordered Pd–Zn Active Pair
Source: Nanomicro Lett. 2024 Jul 15;16:247. doi: 10.1007/s40820-024-01462-w (PMC11250753; doi:10.1007/s40820-024-01462-w)
Supplement: Supplementary file 1 — Supplementary file1 (DOC 14196 KB) [file 40820_2024_1462_MOESM1_ESM.doc]

Supporting Information for

**Boosting Electrochemical Urea Synthesis via Constructing Ordered Pd-Zn Active Pairs**

Weiliang Zhou^1,#^, Chao Feng^1,2,#^, Xuan Li^1^, Xingxing Jiang^1^, Lingyan Jing^1^, Shuai Qi^1^, Qihua Huo^1^, Miaoyuan Lv^1^, Xinbao Chen^1^, Tianchi Huang^1^, Jingwen Zhao^1^, Na Meng^1^, Hengpan Yang^1^, Qi Hu^1^,* and Chuanxin He^1^,*

^1^College of Chemistry and Environmental Engineering, Shenzhen University, Shenzhen, Guangdong 518060, People's Republic of China

^2^School of Chemistry and Chemical Engineering/State Key Laboratory Incubation Base for Green Processing of Chemical Engineering, Shihezi University, Shihezi, Xinjiang, 832003, People's Republic of China

^#^Weiliang Zhou and Chao Feng contributed equally to this work.

*Corresponding authors. E-mail: [hq2016@szu.edu.cn](mailto:hq2016@szu.edu.cn) (Qi Hu); [hecx@szu.edu.cn](mailto:hecx@szu.edu.cn) (Chuanxin He)

**S1 Calculation of Faradaic Efficiency and Yield Rate**

The FE of urea was calculated by the following equation:

FE(%) = (16 × V × c × F) / (60.06 × Q) × 100%

The yield rate of urea was calculated by the following equation:

Yield rate = (c × V) / (m_cat_ × t)

where V is the electrolyte volume, c is the concentration of urea, F is the Faraday constant, Q is the electric quantity, m_cat_ is the loading mass of catalyst, and t is the electrolysis time.

**S2 Ammonia Determination**

The concentration of NH_4_^+^ was determined via the method of indophenol blue. In a typical process, the solution of 1 M sodium hydroxide, 5% salicylic acid, and 5% sodium citrate were served as colorant. Then, 2 mL of colorant, 0.2 mL of 1% mass fraction of sodium nitroferricyanide aqueous solution and 1.0 mL of 0.05 M NaClO were dissolved in 2 mL of electrolyte, then the obtained solution was mixed evenly and reacted in the dark for 2 hours. Finally, the absorbances of each sample were measured at the wavelength of ~662 nm [1]. The standard curve for the quantification of ammonia was displayed in **Fig. S32**.

**S3 Nitrite Determination**

The concentration of NO_2_^-^ was determined via ultraviolet-visible (UV-vis) spectroscopy. Specifically, sulfonic acid (0.5 g) and of N-(1-naphthyl) ethylenediamine dihydrochloride (5 mg) were dispersed in 100 mL deionized water containing 5 mL of acetic acid, and the obtained solution was employed as a colorant. Then, 1 mL of electrolyte and 4 mL of colorant were mixed to form a homogeneous solution. Finally, the absorbance of the solution was detected at the wavelength of ~510 nm [2]. The standard curve for the quantification of nitrite was displayed in **Fig. S33**.

**S4 Determination of H_2_ and CO**

H_2_ and CO were determined by gas chromatography (GC) (Shimadzu GC-2014) equipped with both a flame ionization detector (FID) and thermal conductive detector (TCD). Ar was adopted as the carrier gas for H_2_ and CO quantification.

**S5 Urea Determination**

Urea was first quantified by high performance liquid chromatography (HPLC, Agilent 1260 Infinity II Prime). HPLC was performed on a Luna 5 μm NH_2_ column (250 mm × 4.6 mm). The corresponding mobile phase, flow rate, and detected wavelength were acetonitrile-water (40:60), 0.45 mL min^-1^, and 195 nm, respectively. The standard curve of HPLC for the quantification of urea was shown in Error: Reference source not found.

To accurately quantify the yield rate of urea, diacetyl monoxime and urease decomposition colorimetric method were also carried out. For the approach of modified diacetyl monoxime (M-DAMO-TSC), specifically, 2 g of sulfamic acid was dissolved in 100 mL of water as a NO_2_^-^ elimination reagent. 40 mL of concentrated phosphoric acid, 120 mL of concentrated sulfuric acid and 40 mg of ferric chloride were dispersed in 240 mL deionized water to form a homogeneous solution (solution A), and 2 g of diacetylmonoxime (DAMO) and 40 mg of thiosemicarbazide (TSC) were dissolved in 400 mL of deionized water to form a homogeneous solution (solution B). Then, 1 mL of electrolyte was dispersed in 100 μL of the above reagent and 300 μL 1.0 M HCl to remove NO_2_^-^. Then, 1 mL of electrolyte without NO_2_^-^ dispersed in 2 mL of solution A and 1 mL of solution B, and the mixture was placed in a boiling water bath for 25 min. After cooling down to room temperature, the absorbance of the samples was acquired at ~525 nm using a UV‒vis spectrophotometer (Shimadzu GC-2550). The standard curve of DAMO-TSC for the quantification of urea was shown in Error: Reference source not found**.** For the approach of urease decomposition, according to the previous literature, 3.6 mL of urea electrolyte was mixed with 0.5 mL of urease solution containing a concentration of 5 mg mL^-1^. The mixture was kept in a constant temperature shaker at 37 ℃ for 1h. During this period, urea ((NH_2_)_2_CO) was broken down by urease into two NH_3_ molecules and CO_2_. The NH_3_ concentration in the electrolyte solution containing urease was measured using the indophenol blue method. For comparison, NH_3_ concentration in urea electrolyte without urease was also measured. The total moles of ammonia (m_urease_) in the electrolyte were determined using a spectrophotometer, which were expressed as 2m_urea_+m_ammonia_. In this equation, 2m_urea_ represents the moles of ammonia produced by the urease-caused decomposition. Thus, the number of moles of urea produced (m_urea_) was calculated by (m_urease_-m_ammonia_) / 2 [S1-S3].

**S6 Isotope Tracer Experiments of ^15^N**

The urease decomposition method was implemented to conduct an isotope tracer experiment using ^15^N, and ^15^N in the electrolyte of K^15^NO_3_ was measured before and after urea decomposition. Specifically, 500 uL solution containing 1mL 1mg mL^-1^ maleate (Internal standard), 0.5 mL 4 M H_2_SO_4_ and 1 mL electrolyte, then 100 uL were dispersed in the solution. The data was collected via 256 scans conducted on a 500 MHz NMR instrument (Bruker) equipped. Additionally, the content of ^14^N in the electrolyte of K^14^NO_3_ were measured before and after urea decomposition, corresponding results were presented in **Fig. S14**.

**S7 Operando Differential Electrochemical Mass Spectrometry (DEMS) Measurements**

The intermediate products were detected by DEMS (QAS100). In the coreduciton reaction of CO_2_ and NO_3_^-^, the electrolyte was 0.1 M KNO_3_ and 0.2 M KHCO_3_ solution, and Ar bubbled into the electrolyte before and during DEMS measurement. The signals were collected under the potential of -0.4 V vs. RHE electrolysis conditions.

**S8 Electrochemical Operando ATR-FTIR Measurements**

The Si prisms were polished with an Al_2_O_3_ suspension and sequentially ultrasonically cleaned in a bath of acetone and deionized water before depositing a certain thickness of Au onto the surface of the Si prism. The prepared catalyst ink was loaded onto the above prepared Au film to prepare the working electrode. ATR-SERAS measurements were performed in a two-compartment spectroscopic electrochemical cell containing three electrodes, including a working electrode, a standard Ag/AgCl electrode as a reference electrode, and a platinum wire as a counter electrode, respectively. All ATR-SERAS spectra were acquired using a Fourier transform infrared spectrophotometer (FT-IR, Nicolet Is50 Thermo Fischer Scientific) equipped with a mercury cadmium telluride (MCT) detector. Electrochemical tests were performed in 0.1 M KNO_3_ and 0.2 M KHCO_3_ aqueous solution with constant CO_2_ flow and controlled by a CHI electrochemical workstation (CHI660E). In a typical test, the working electrode was initially activated by running CV cycles between 0 and -1 V vs. RHE at a scan rate of 0.05 V s^-1^ until the system was stabilized. Then, the spectrum was collected under the open-circuit potential as the background signal, and the cathodic potential was adjusted from -0.2 to -0.6 V vs. RHE with an interval of 0.1 V. Each potential lasted 2 min for spectral acquisition.

**S9 Theoretical Calculation Method**

All the calculations are performed in the framework of the density functional theory with the projector augmented plane-wave method, as implemented in the Vienna ab initio simulation package [S5]. In this work, all calculations were carried out based on the VASP code. The generalzied gradient approximation proposed by Perdew, Burke, and Ernzerhof is selected for the exchange-correlation potential [S6]. The cut-off energy for plane wave is set to 550 eV. The energy criterion is set to 10^-5^ eV in iterative solution of the Kohn-Sham equation. A vacuum layer of 10 Å is added perpendicular to the sheet to avoid artificial interaction between periodic images. The Brillouin zone integration is performed using a 3×3×1 k-mesh. All the structures are relaxed until the residual forces on the atoms have declined to less than 0.02 eV/Å. The bottom two layers were fixed during the calculation process. The Gibbs free energy change of each elementary step can be calculated by Δ*G* = Δ*E* + Δ*E*_ZPE_ - *T*Δ*S*. The climbing image nudged elastic band (cNEB) method was used to search the reaction path and transition state, and the vibration frequency calculation was used to confirm it further [S7].

**S10 Supplementary Figures and Tables**


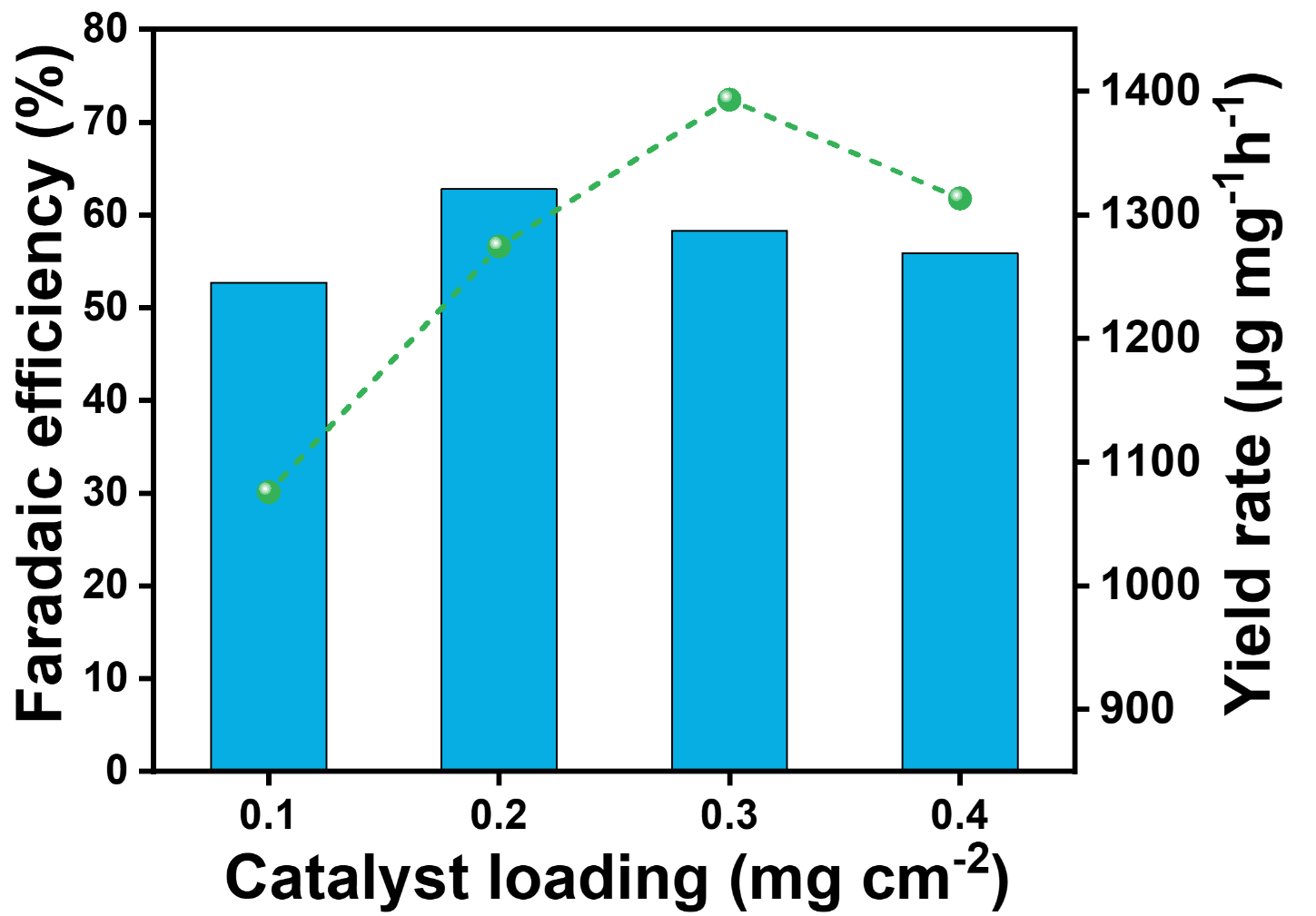


**Fig. S1** Faradaic efficiency and yield rate of urea with different catalyst loadings


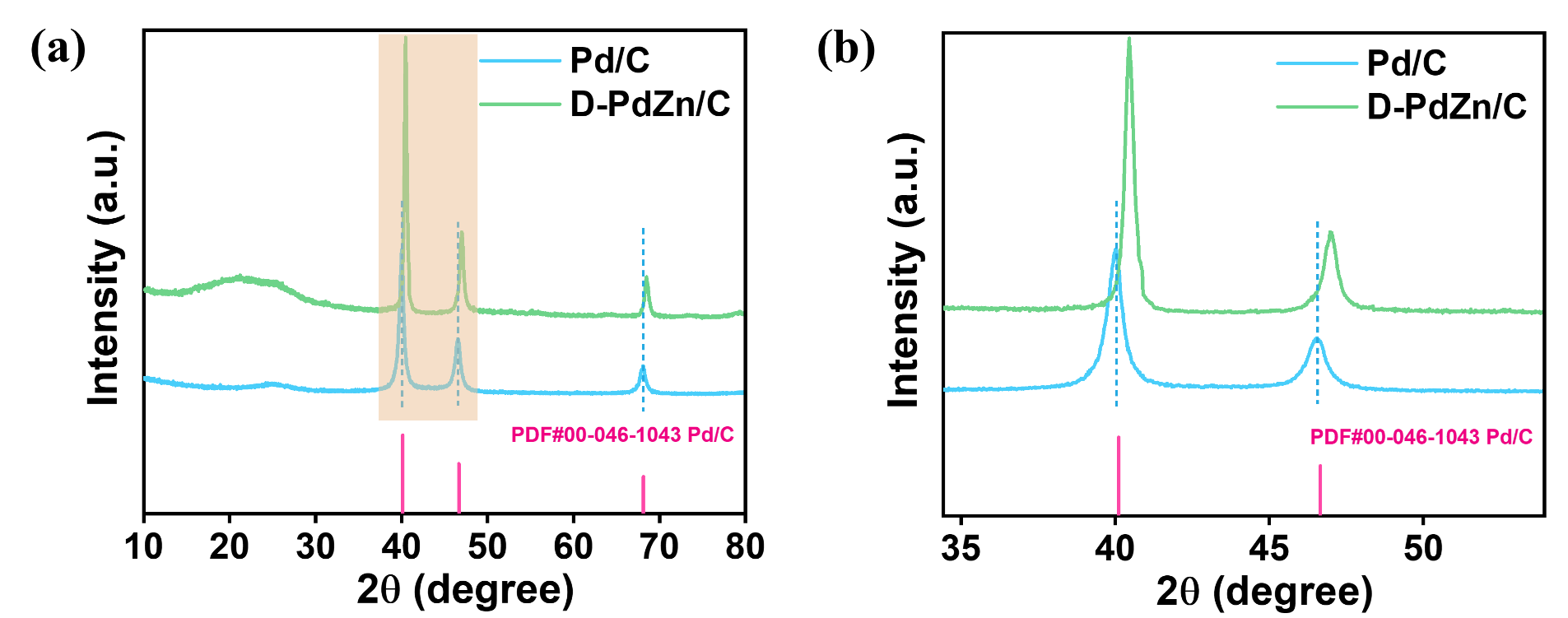


Fig. S2 XRD pattern of Pd/C and D-PdZn/C


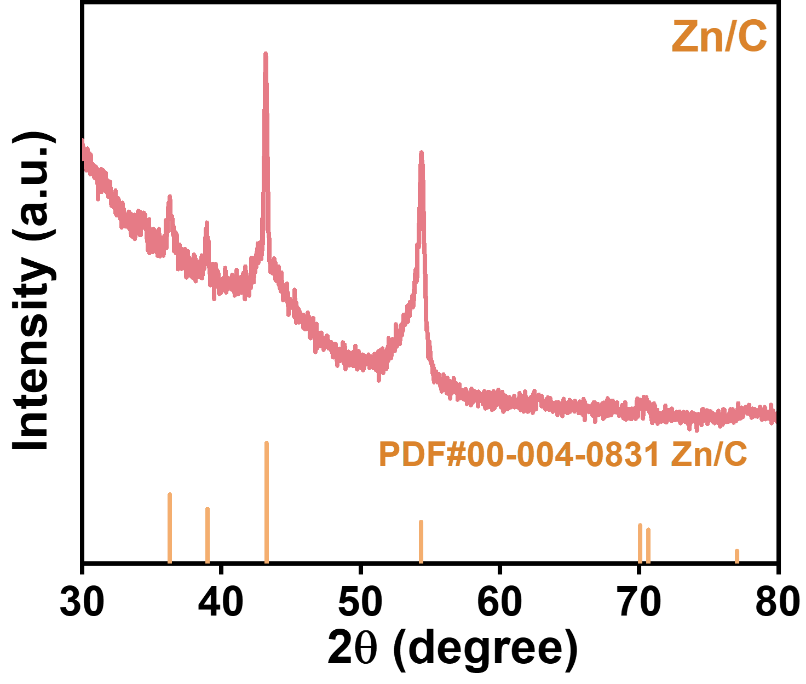


Fig. S3 XRD pattern of Zn/C


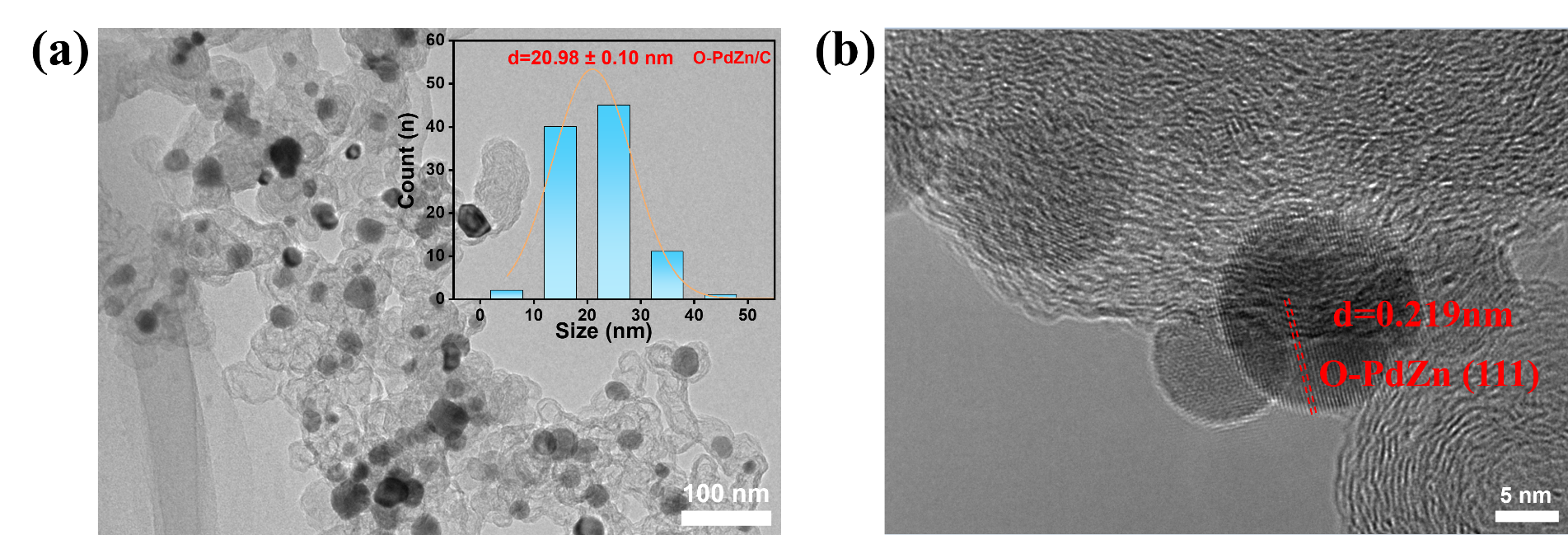


Fig. S4 (a) TEM and size distribution of O-PdZn/C, and (b) HR-TEM of O-PdZn/C


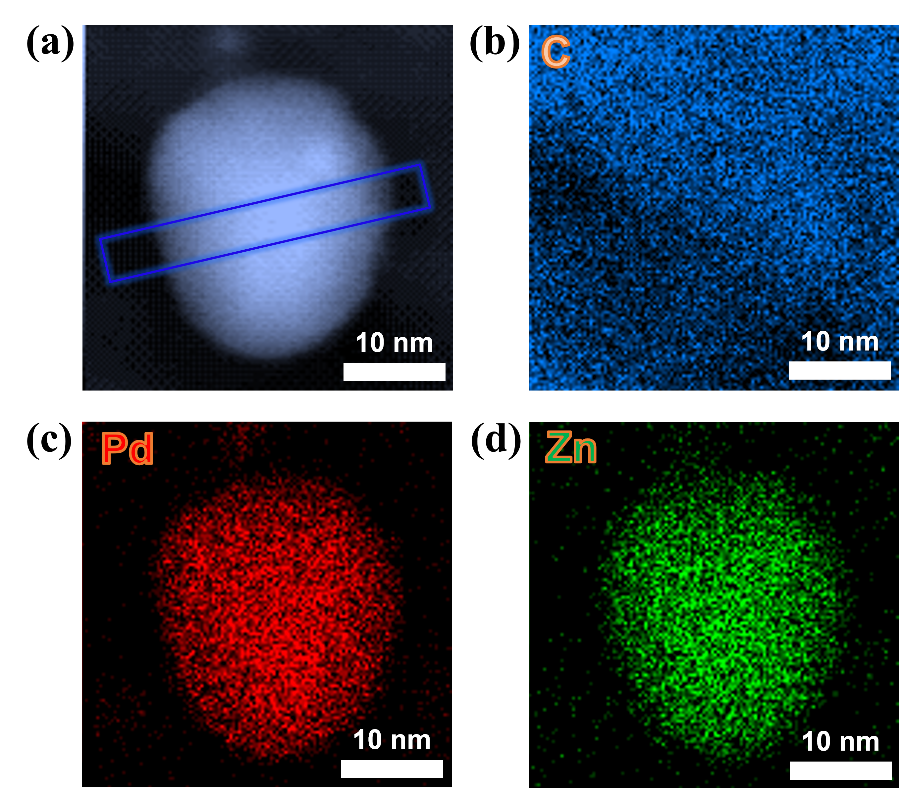


Fig. S5 TEM and EDX element mapping images of O-PdZn /C before reaction

**Table S1** Mass percentage of metal content determined by ICP-OES

| **Metal content** | | |
| --- | --- | --- |
| Sample | Pd mass percentage (%) | Zn mass percentage (%) |
| O-PdZn/C | 7.23 | 7.19 |


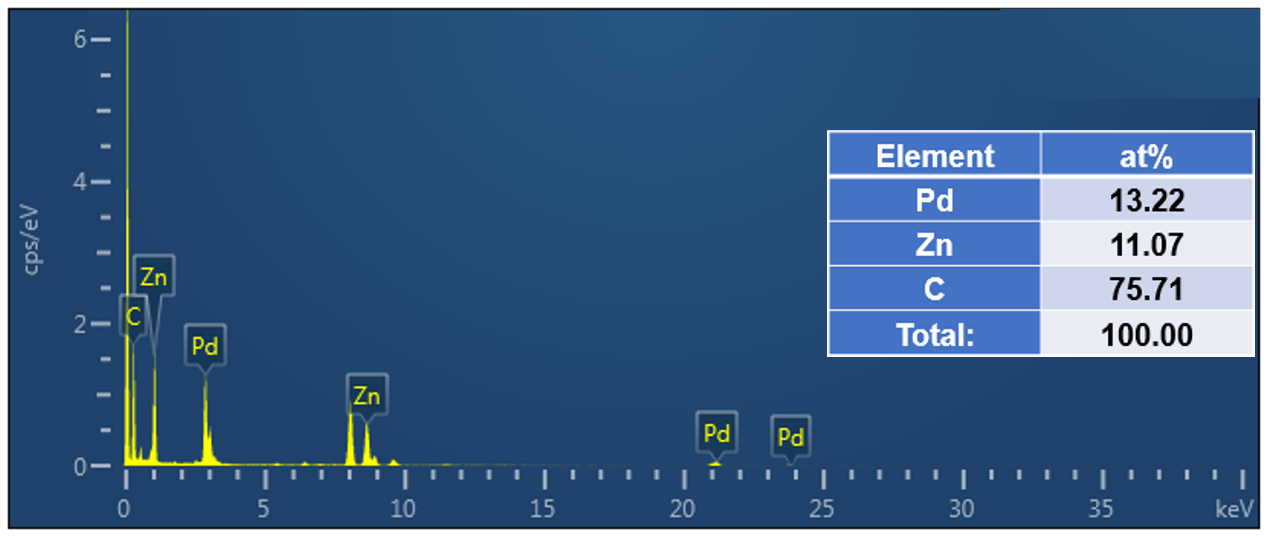


Fig. S6 EDX element distribution image of O-PdZn/C


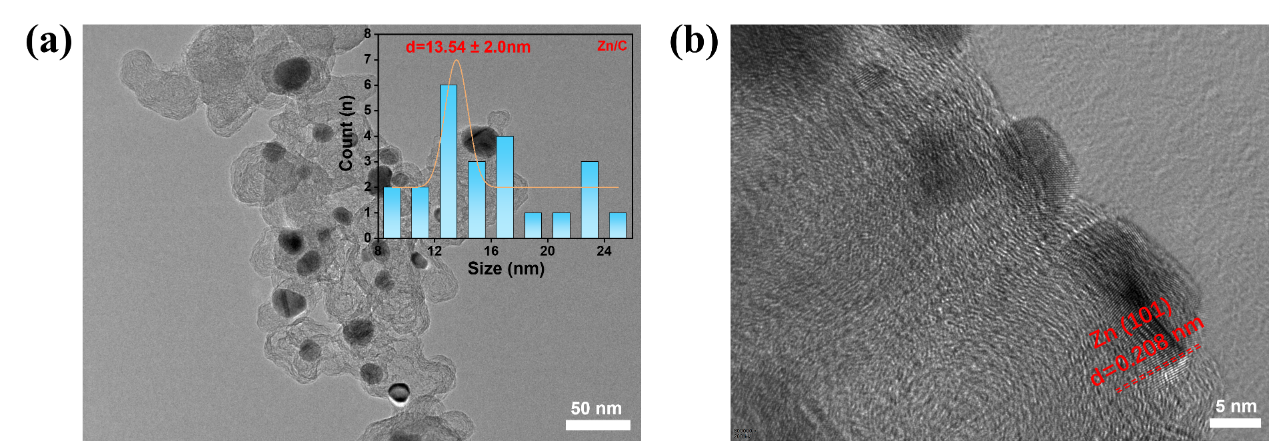


Fig. S7 (a) TEM and size distribution of Zn/C, and (b) HR-TEM of Zn/C


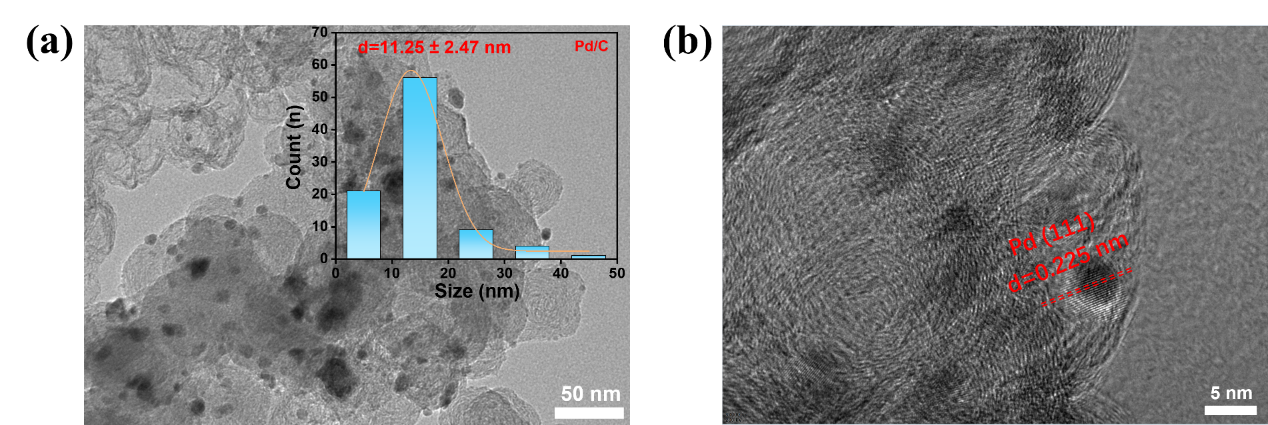


Fig. S8 (a) TEM and size distribution of Pd/C, and (b) HR-TEM of Pd/C


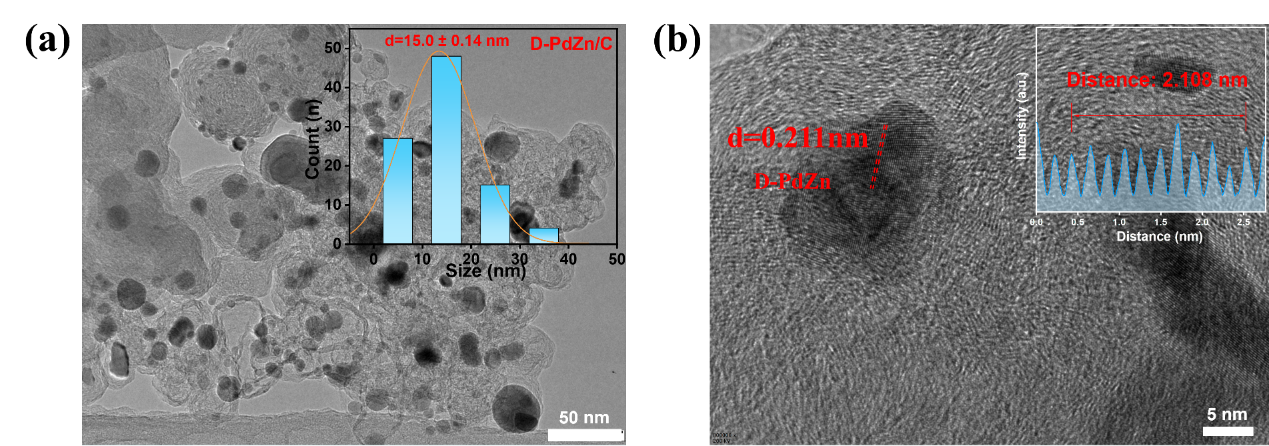


Fig. S9 (a) TEM and size distribution of D-PdZn/C, and (b) HR-TEM of D-PdZn/C


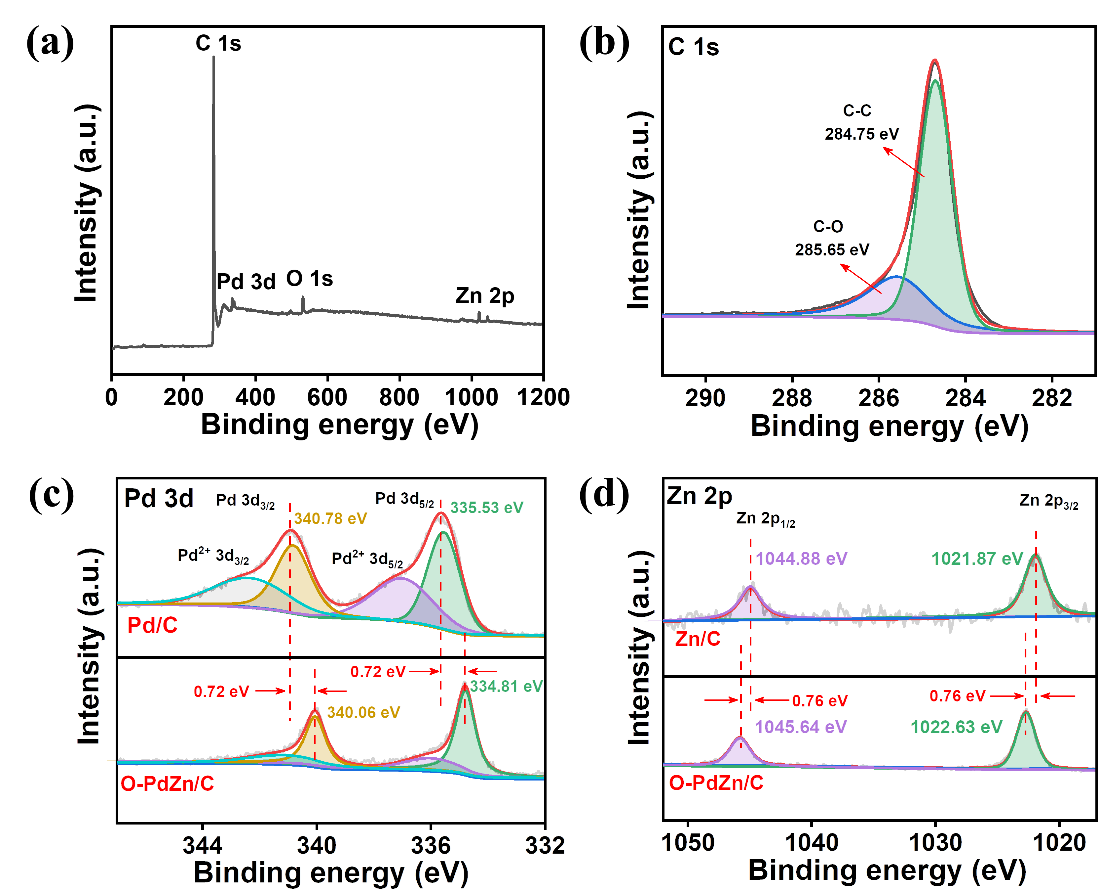


Fig. S10 (a) XPS survey spectra, (b) C 1s, (c) Pd 3d, and (d) Zn 2p XPS spectrum of O-PdZn/C


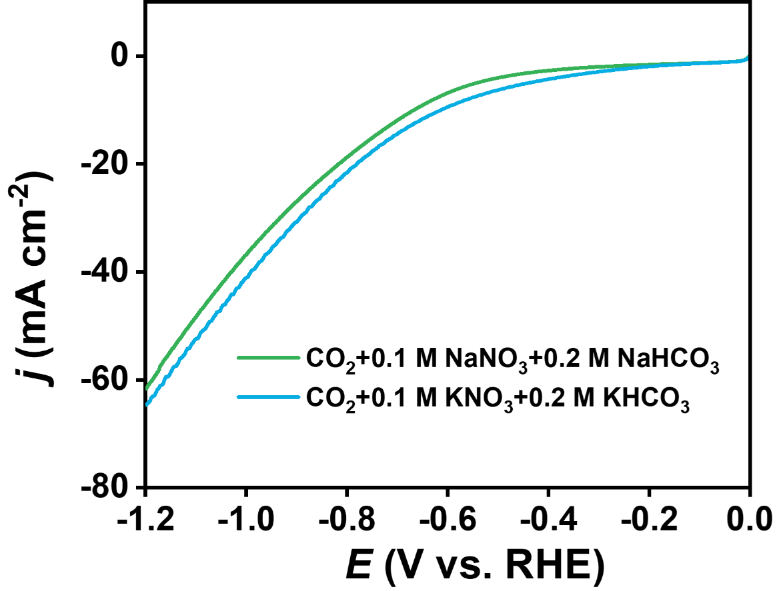


**Fig. S11** LSV curves of O-PdZn/C for the co-reduction of NO_3_^-^ and CO_2_ in different electrolytes


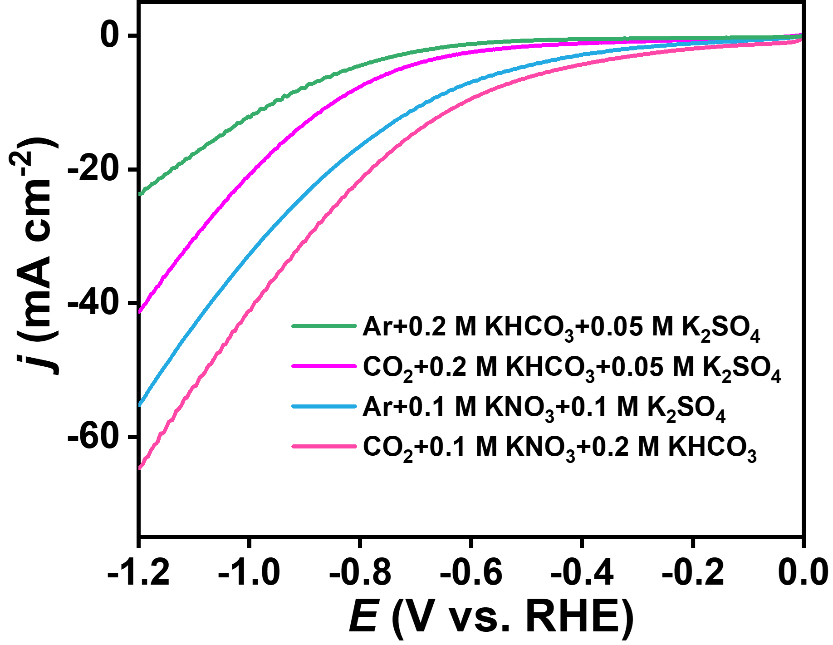


Fig. S12 LSV curves of O-PdZn/C without CO_2_ and NO_3_^–^, with individual CO_2_, individual NO_3_^-^ and coexisting CO_2_ and NO_3_^–^


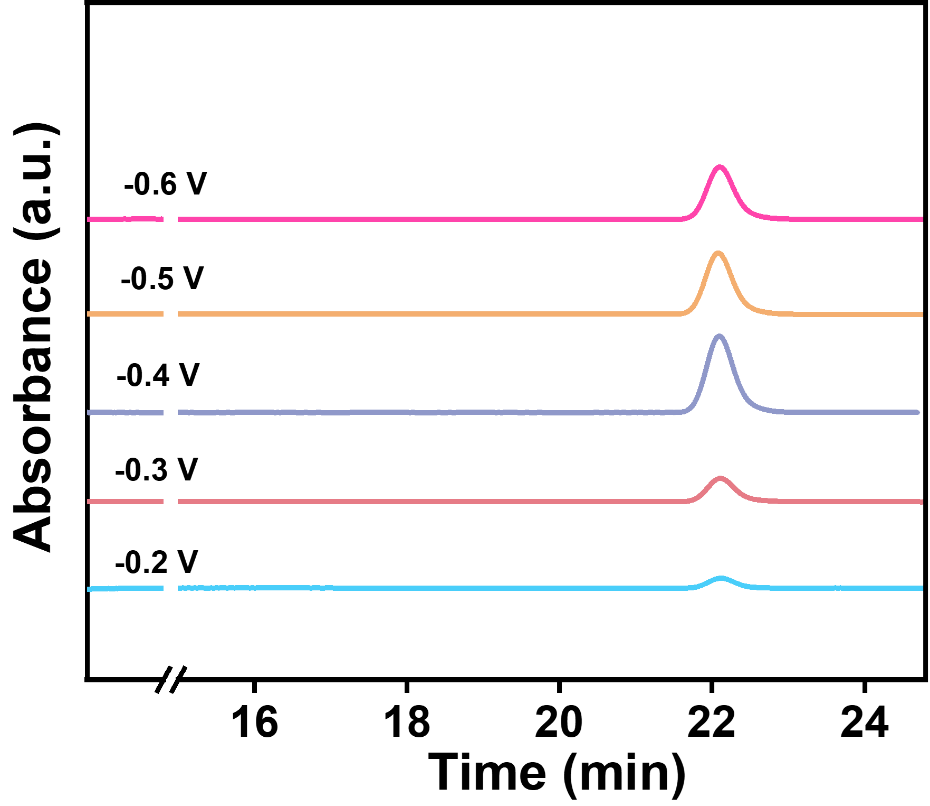


Fig. S13 HPLC profile of urea content under different potentials

**Fig. S14** The partial current density of urea (*j*_urea_) for various catalysts at various potential


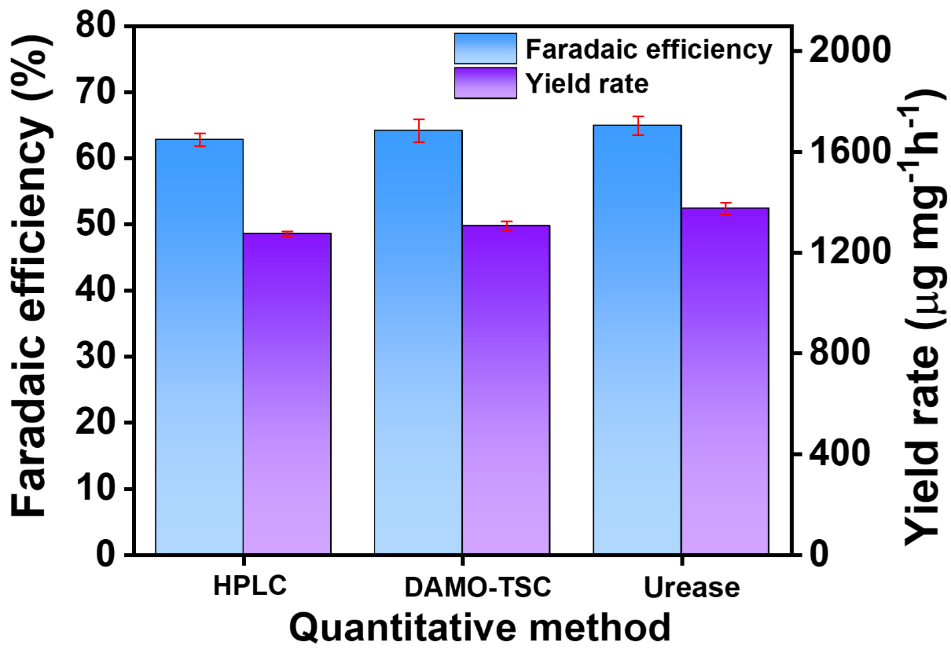


Fig. S15 Faradaic efficiency and yield of different quantitative urea methods


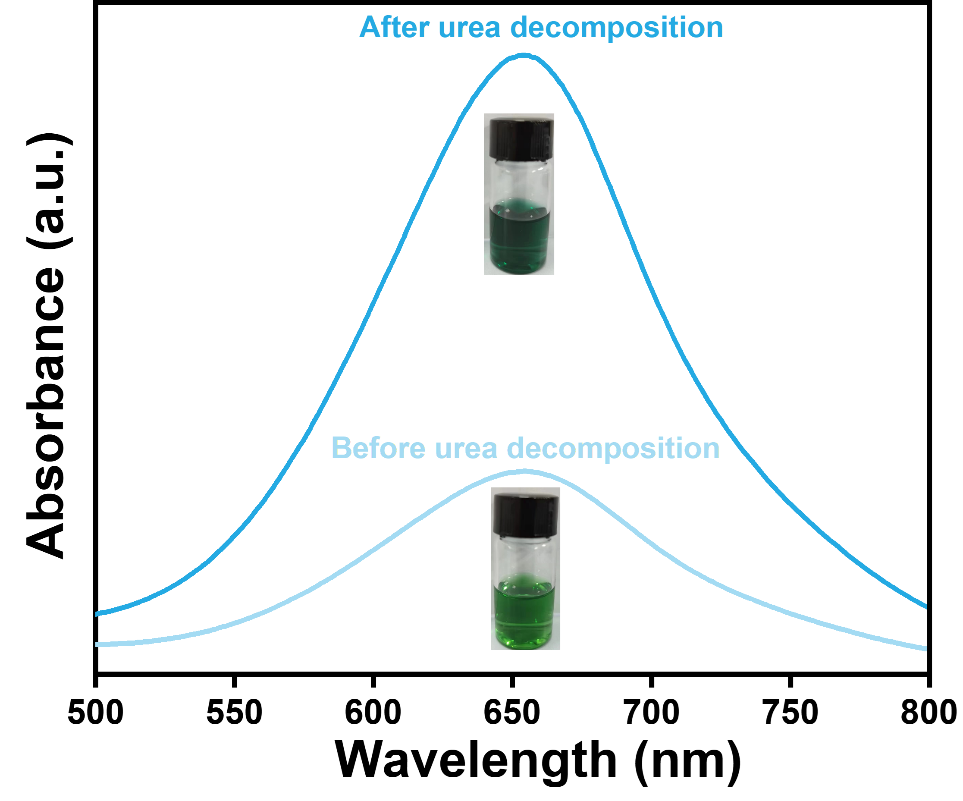


Fig. S16 UV-vis absorption spectra of NH_4_^+^ concentrations before and after urea decomposition at –0.4 V


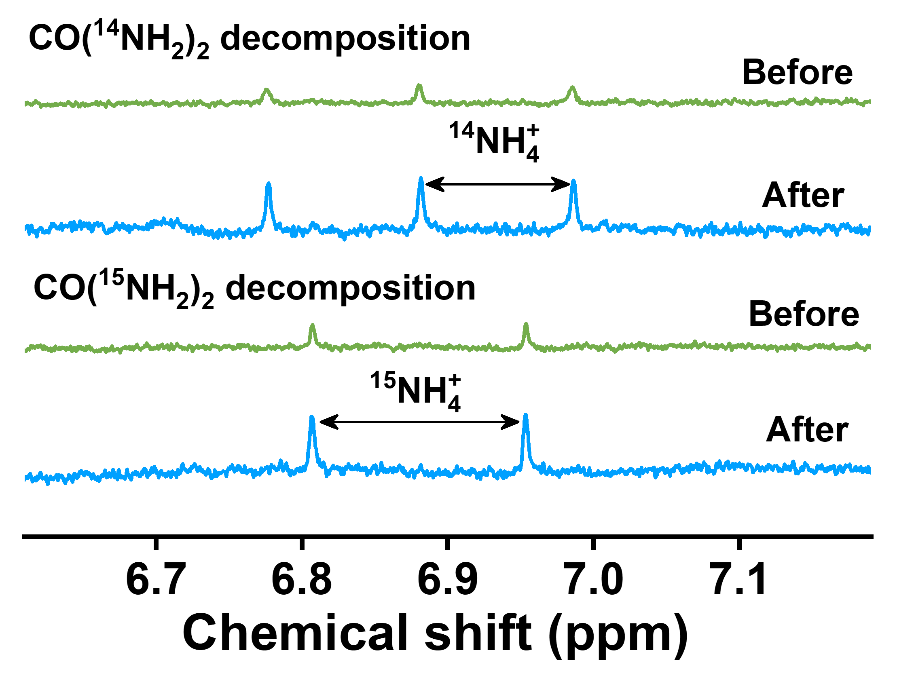


Fig. S17 ^1^H NMR spectra of ^14^NH_4_^+^ and ^15^NH_4_^+^ before and after decomposition of urea products on O-PdZn/C at –0.4 V vs RHE


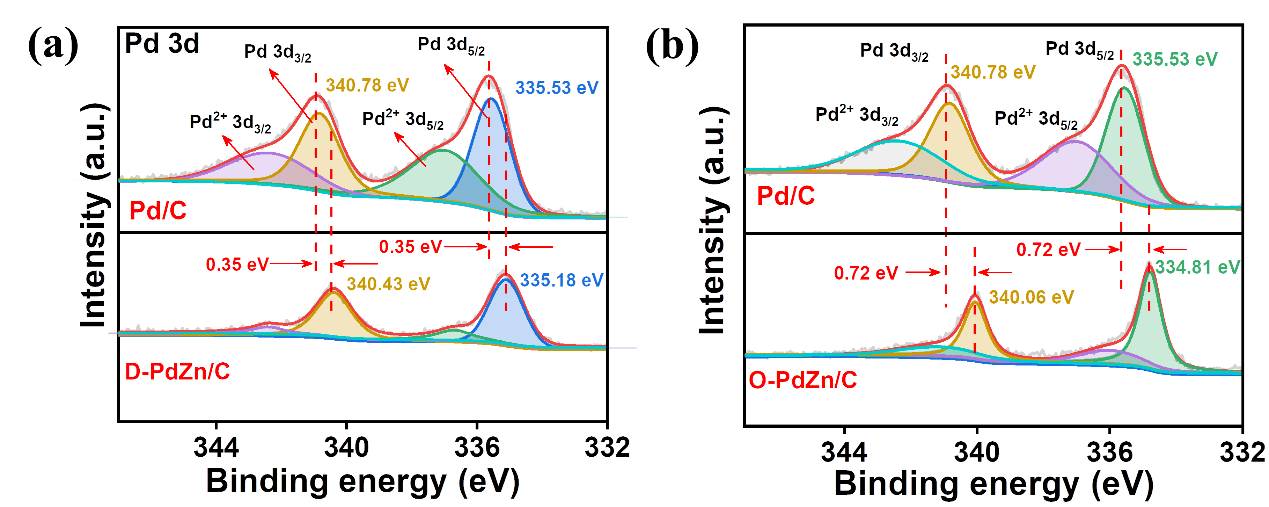


Fig. S18 Pd 3d XPS spectrum of (a) Pd/C and D-PdZn/C, (b) Pd/C and O-PdZn/C


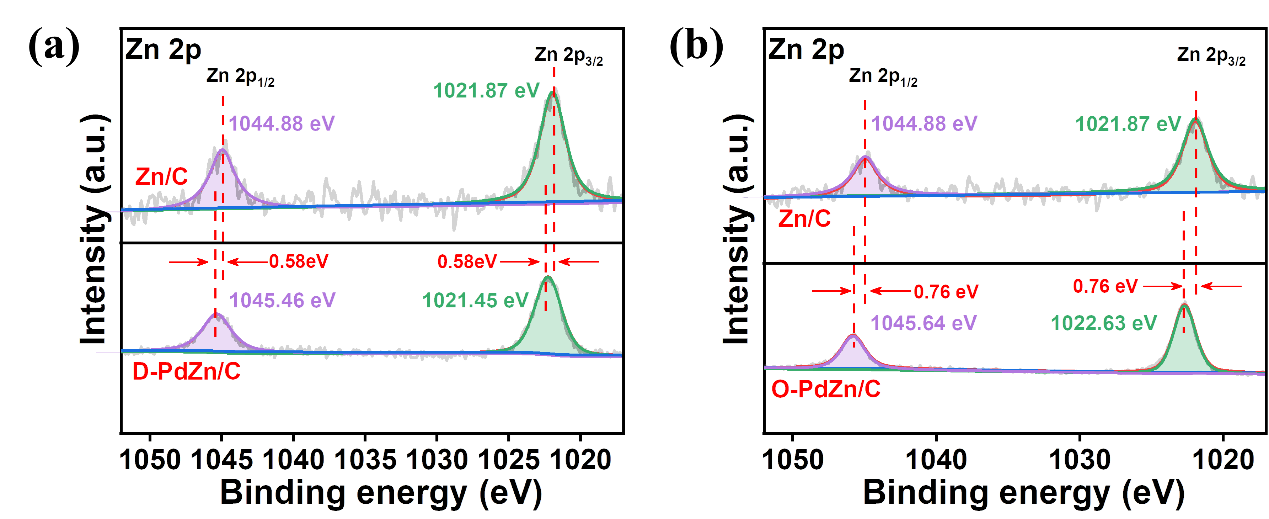


Fig. S19 Zn 2p XPS spectrum of (a) Zn/C and D-PdZn/C Zn 2p, (b) Zn/C and O-PdZn/C


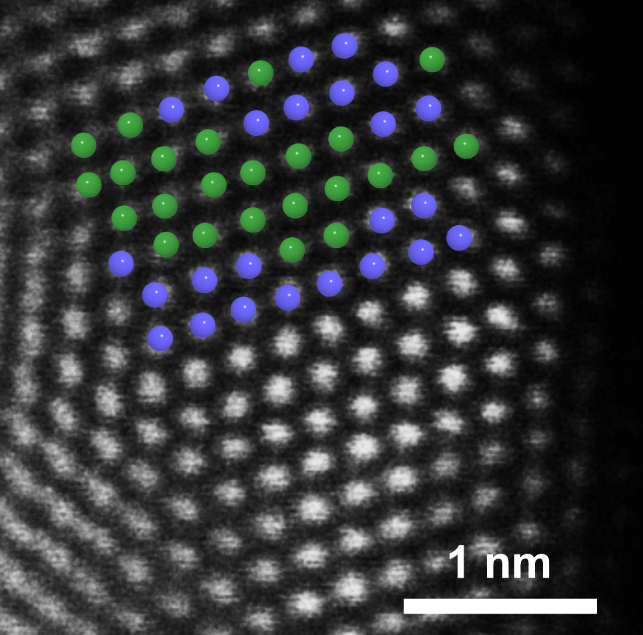


**Fig. S20** The HAADF STEM image of D-PdZn/C


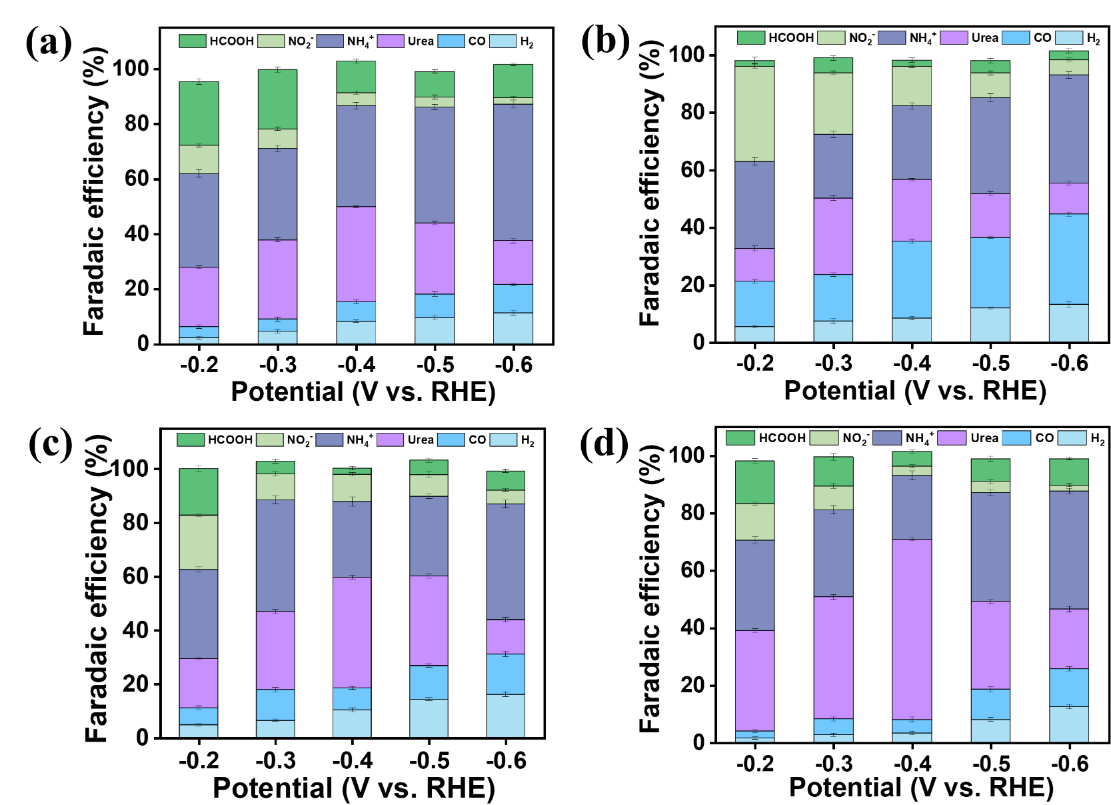


**Fig. 21** Product distribution of various catalyst


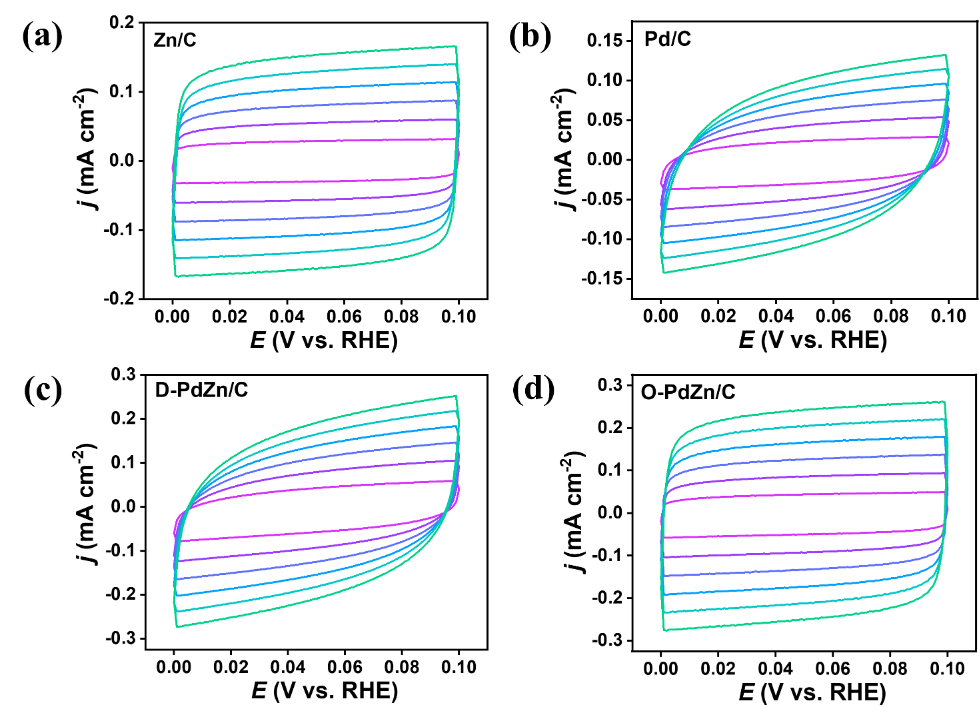


Fig. S22 CV curves of various catalysts


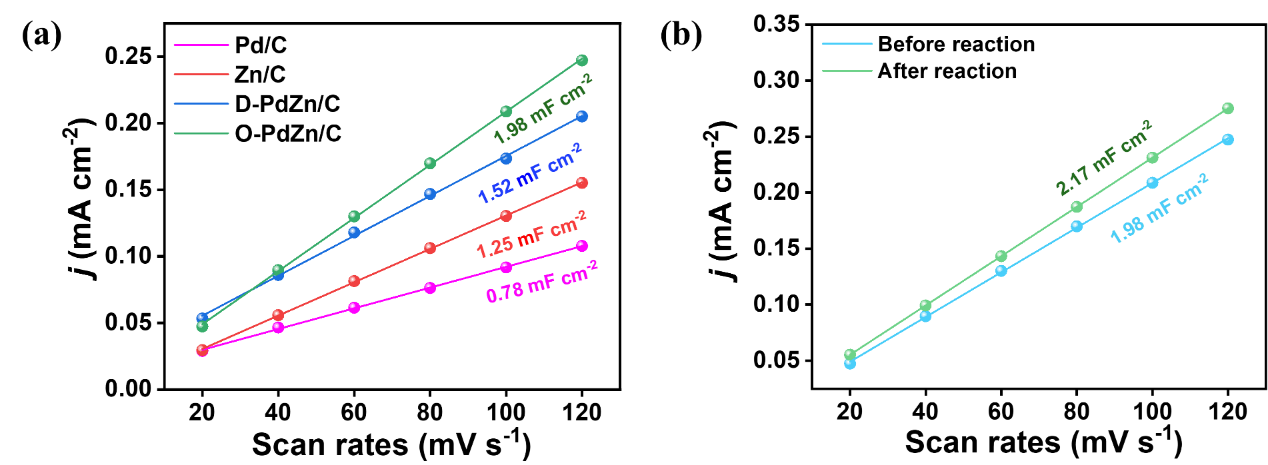


Fig. S23 (a) C_dl_ value of different catalysts, (b) C_dl_ value of O-PdZn/C before and after reaction

Fig. S24 The yield rate of urea normalized by C_dl_ values


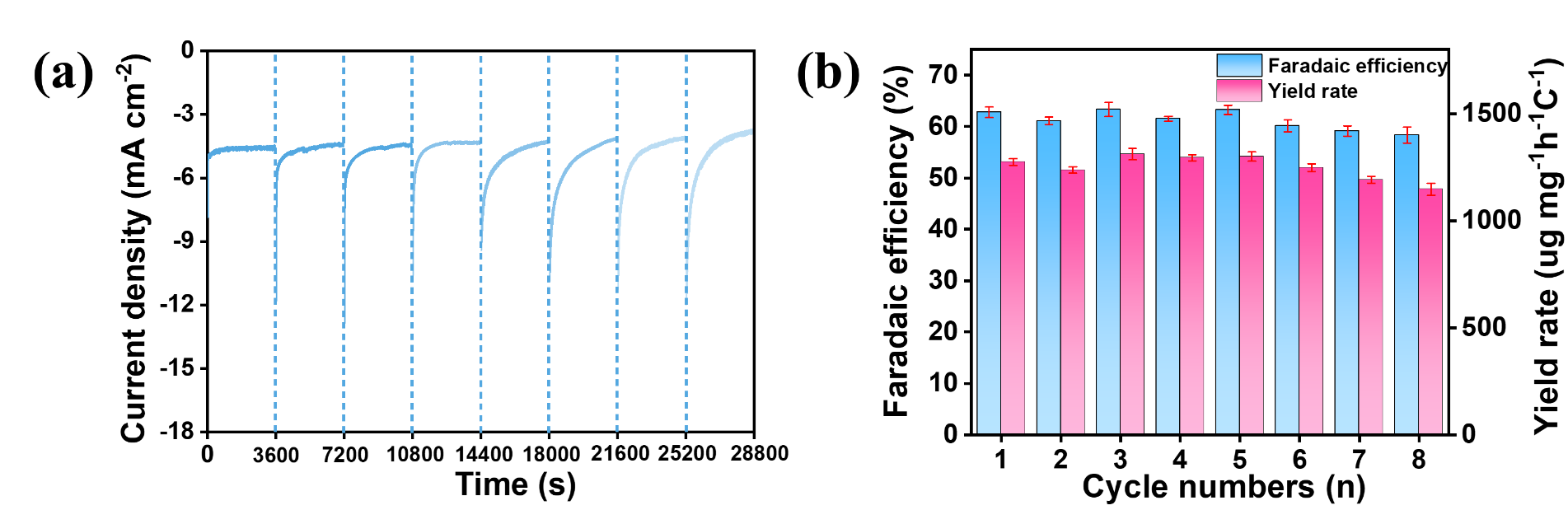


Fig. S25 (a) I-T curves profile, (b) Faradaic efficiency and yield of continuous urea synthesis for eight cycles at –0.4 V vs RHE


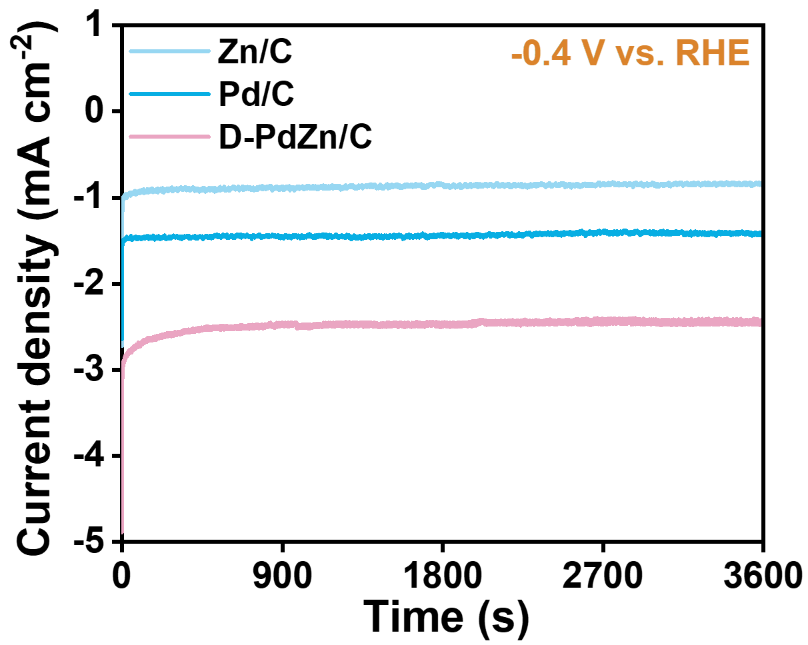


Fig. S26 I-t curves profile of different catalysts


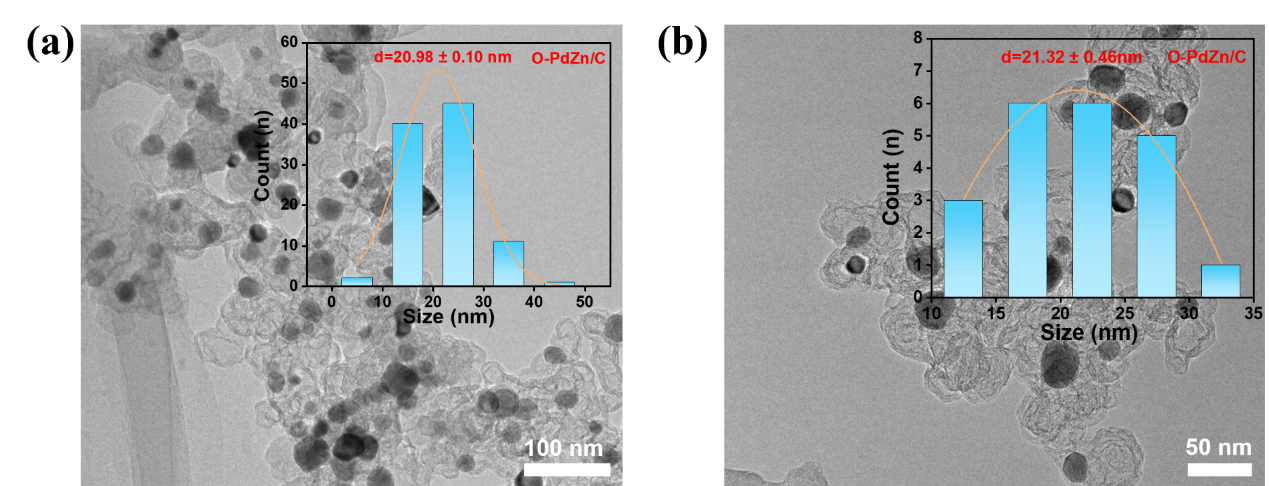


Fig. S27 TEM images of O-PdZn/C before and after reaction


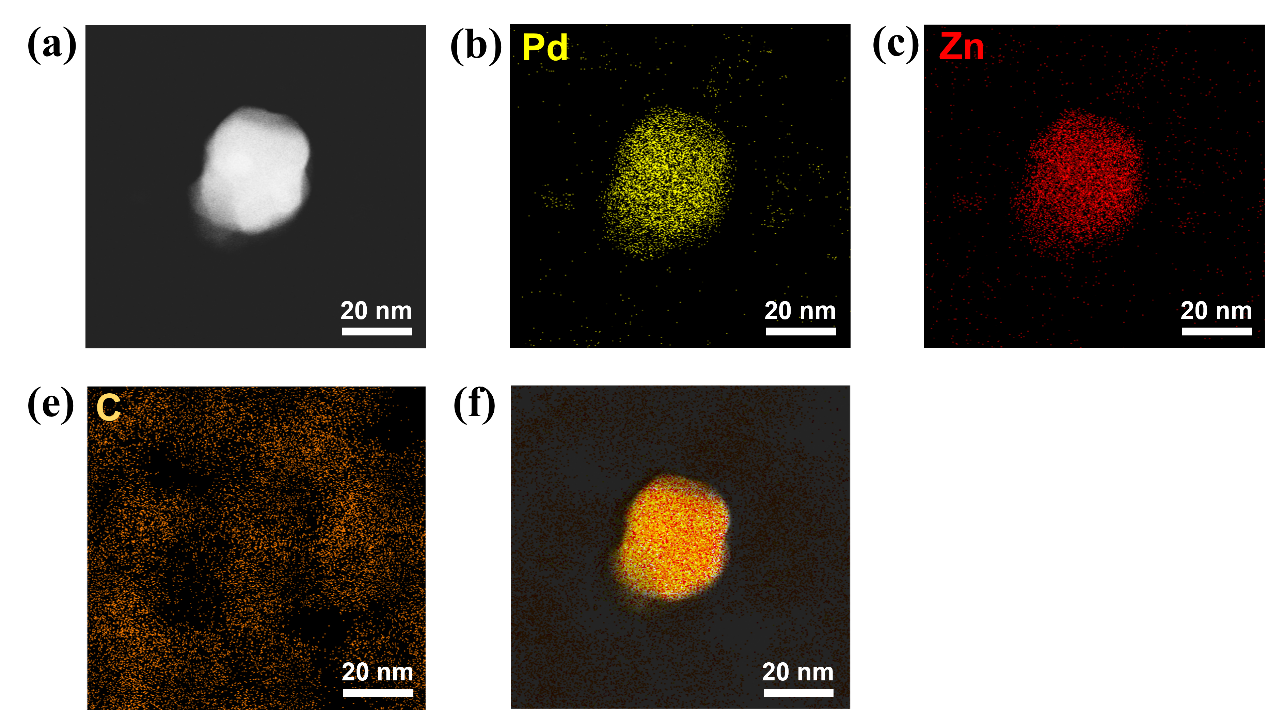


Fig. S28 EDX elements mapping of O-PdZn/C after reaction


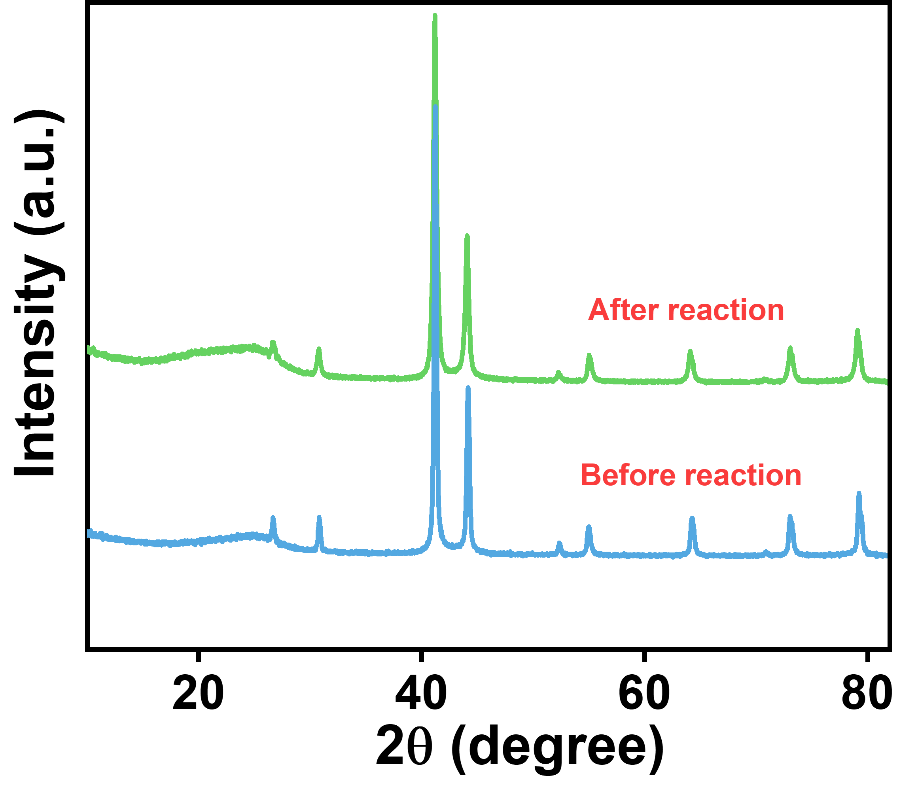


Fig. S29 XRD pattern of O-PdZn/C before and after reaction


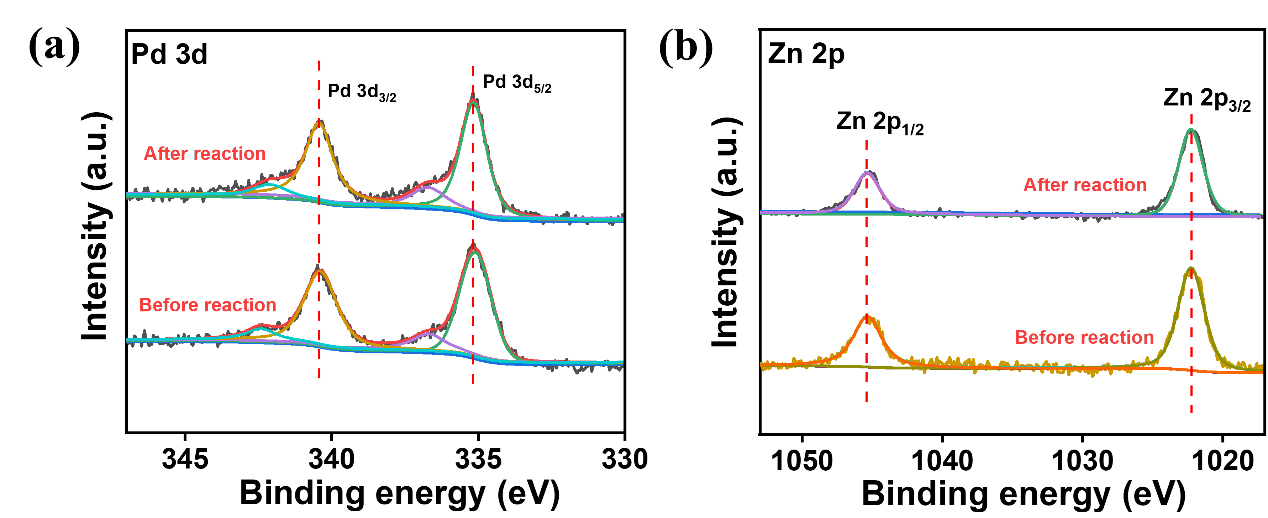


Fig. S30 (a) Pd 3d, (b) Zn 2p XPS spectrum of O-PdZn/C before and after reaction


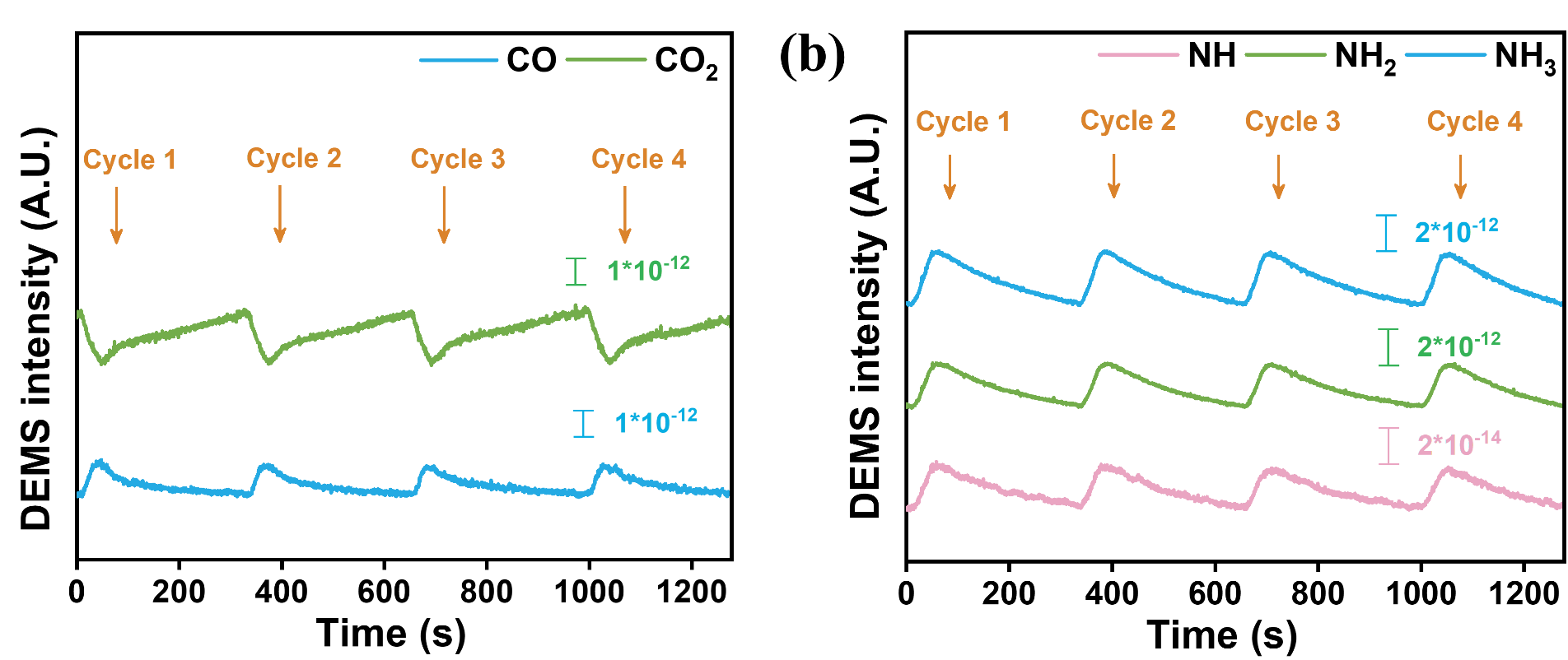


Fig. S31 DEMS signal of O-PdZn/C at –0.4 V vs. RHE

**Fig. S32** Operando FTIR signal of H_2_O for various catalyst


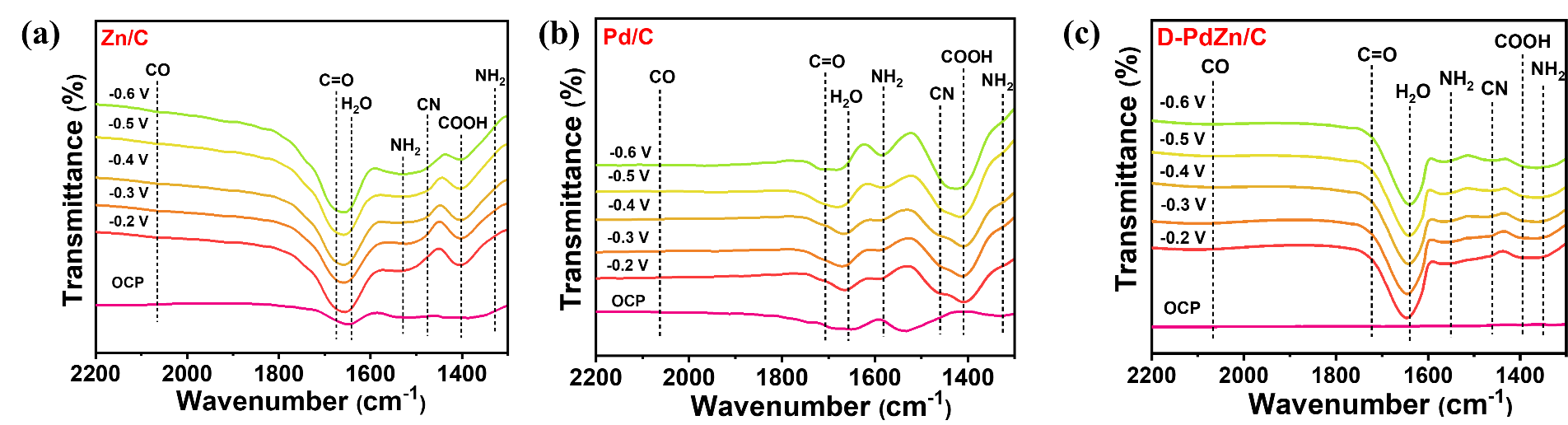


Fig. S33 Operando FTIR of Zn/C, Pd/C and D-PdZn/C in the range of 1300-2200 cm^–1^


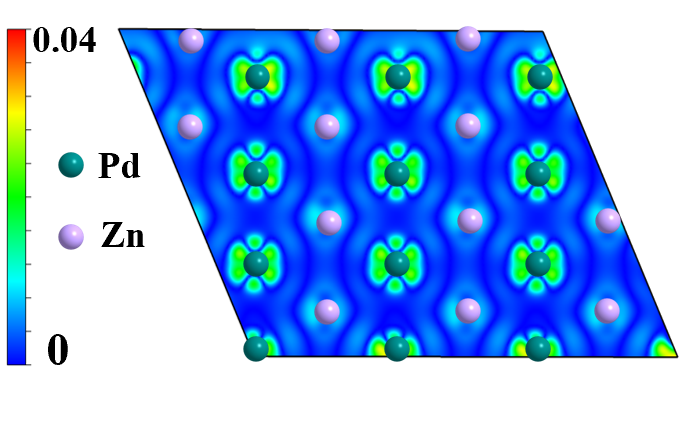


Fig. S34 The differential charge density maps of O-PdZn/C


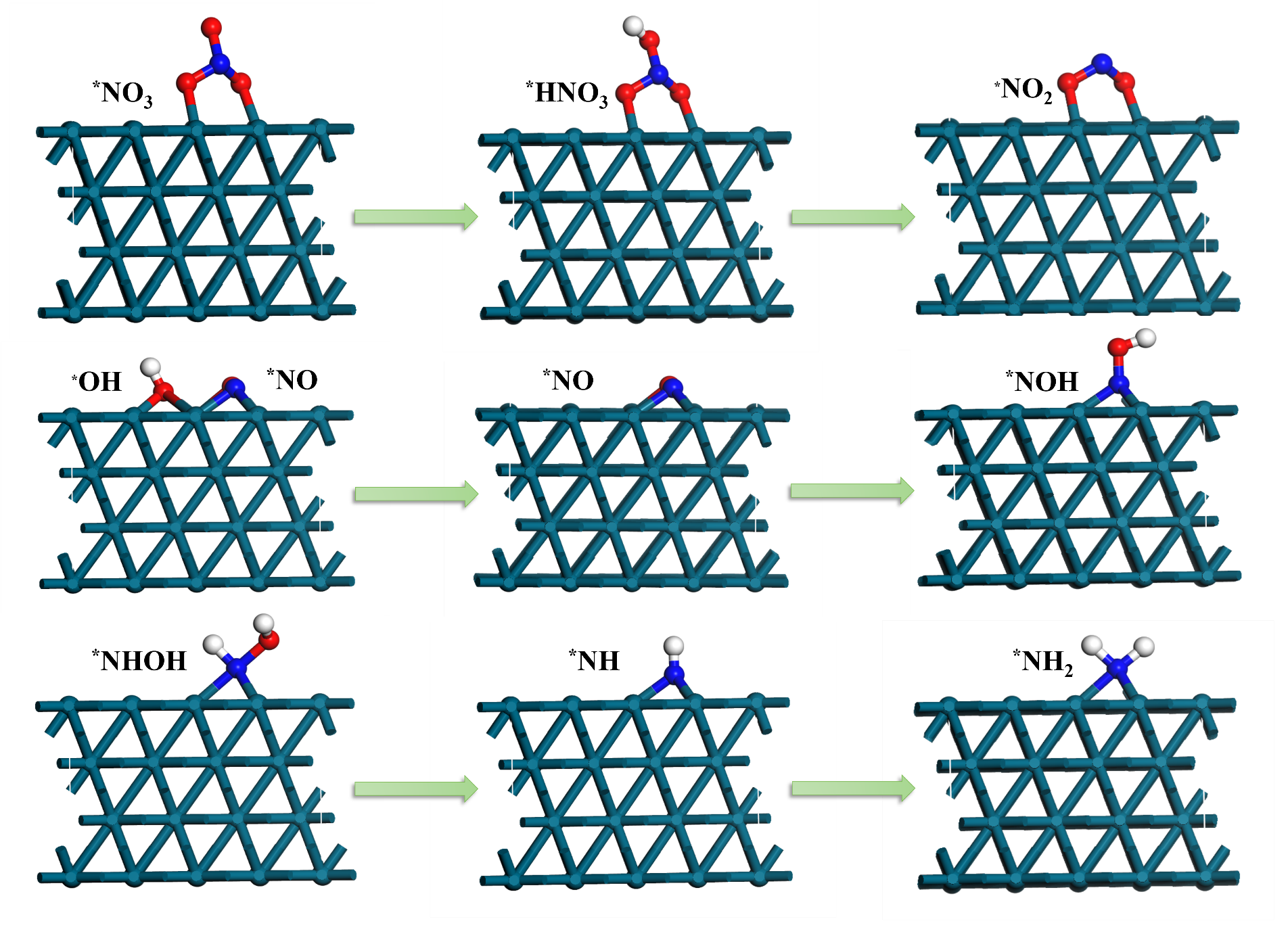


Fig. S35 The atomic models of all the intermediates adsorption on the surface of Pd/C for *NO_3_ reduction to *NH_2_


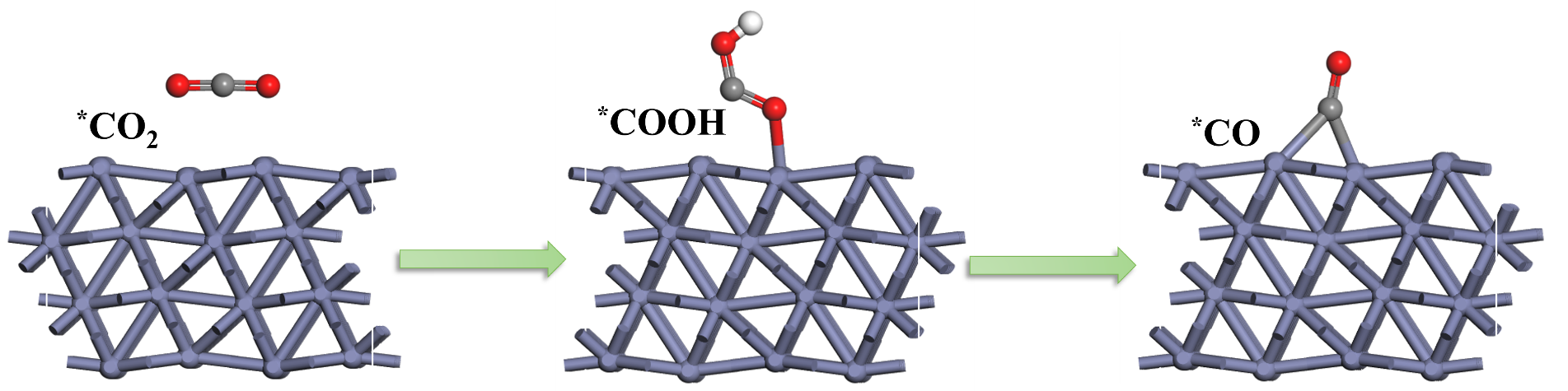


Fig. S36 The structural models of all the intermediates adsorption on the surface of Zn/C for *CO_2_ reduction to *CO


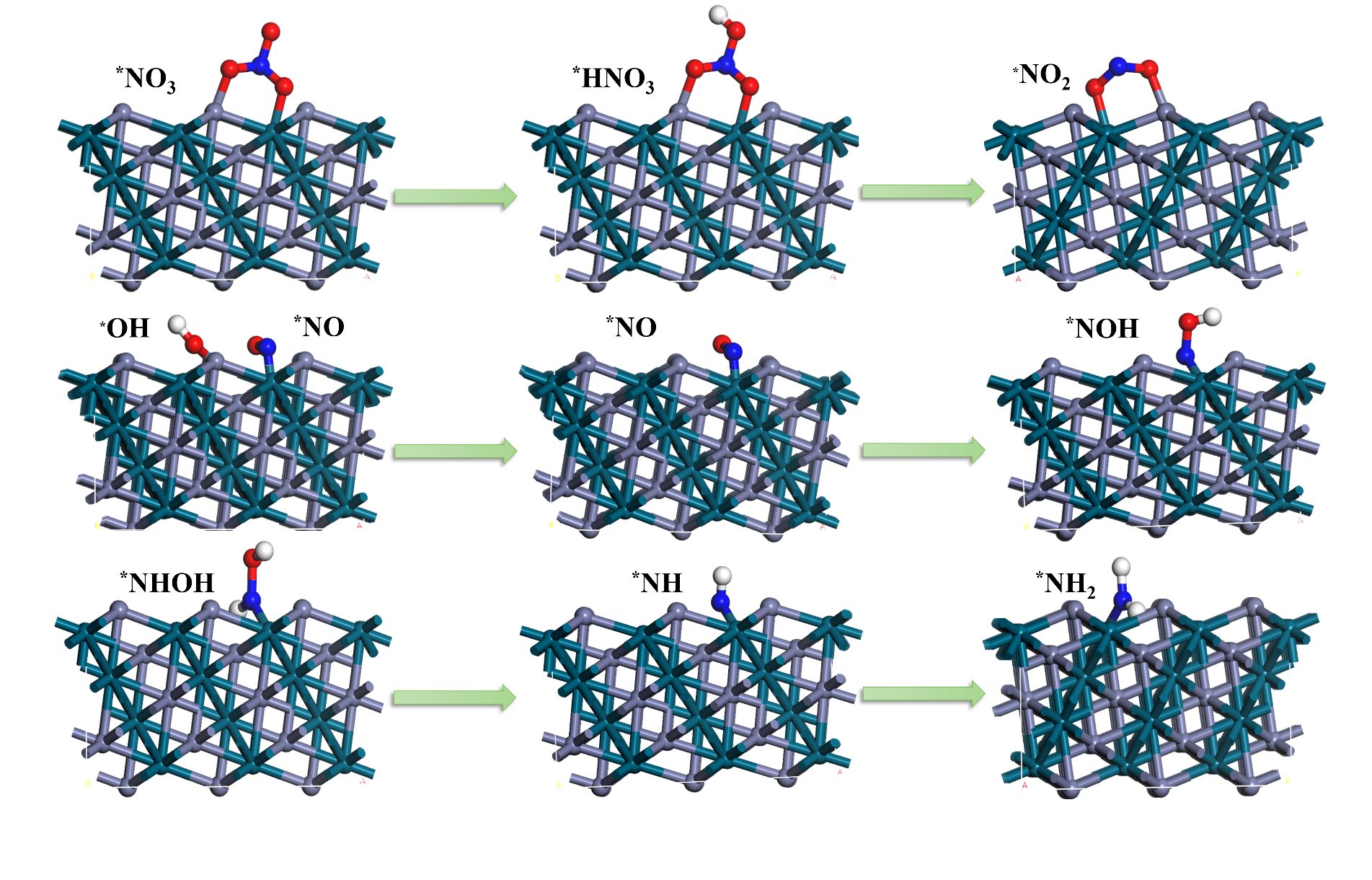


Fig. S37 The structural models of all the intermediates adsorption on the surface of O-PdZn/C for *NO_3_ reduction to *NH_2_


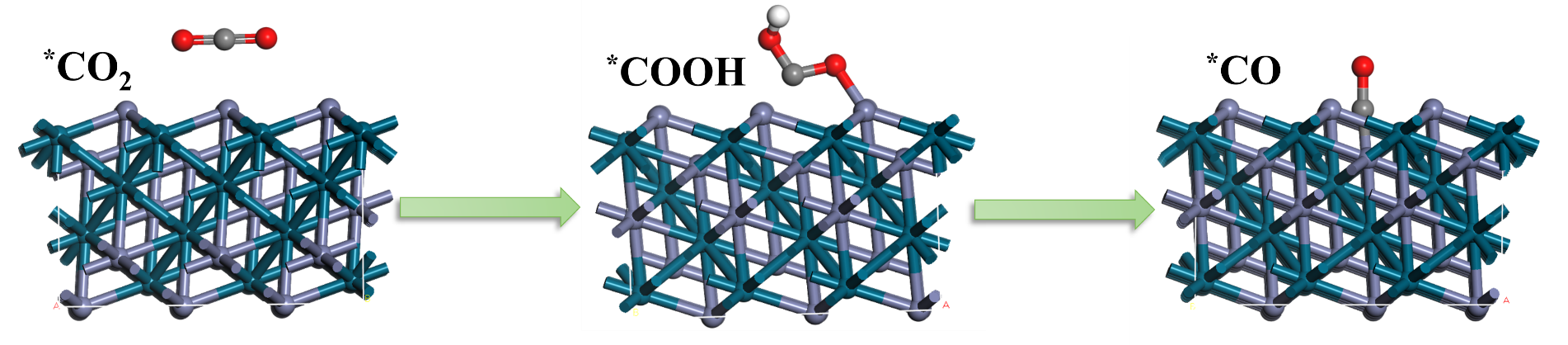


Fig. S38 The structural models of all the intermediates adsorption on the surface of O-PdZn/C for *CO_2_ reduction to *CO


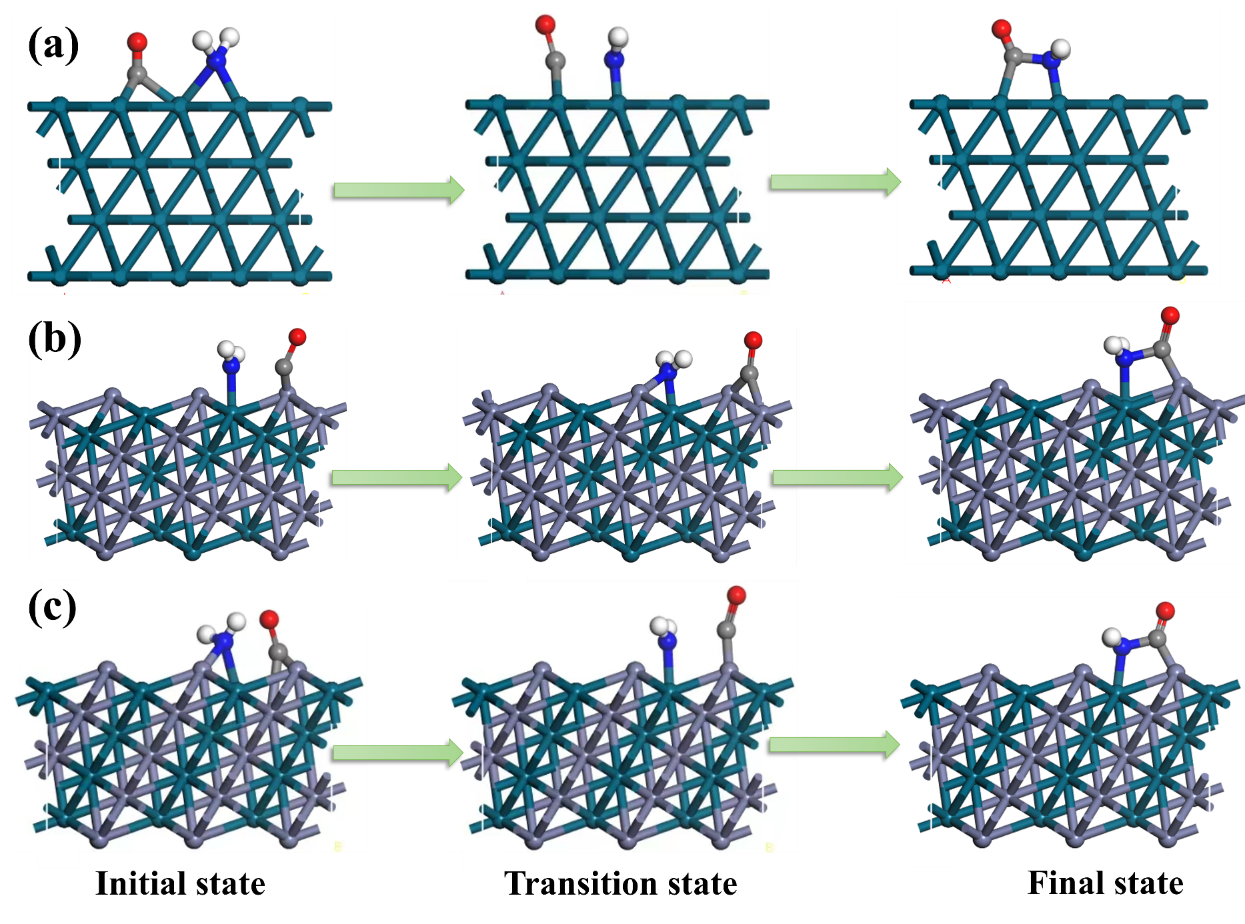


Fig. S39 The structures of the initial, transition and final state for *CONH_2_ formation on the surface of (a) Pd/C, (b) D-PdZn/C, and (c) O-PdZn/C


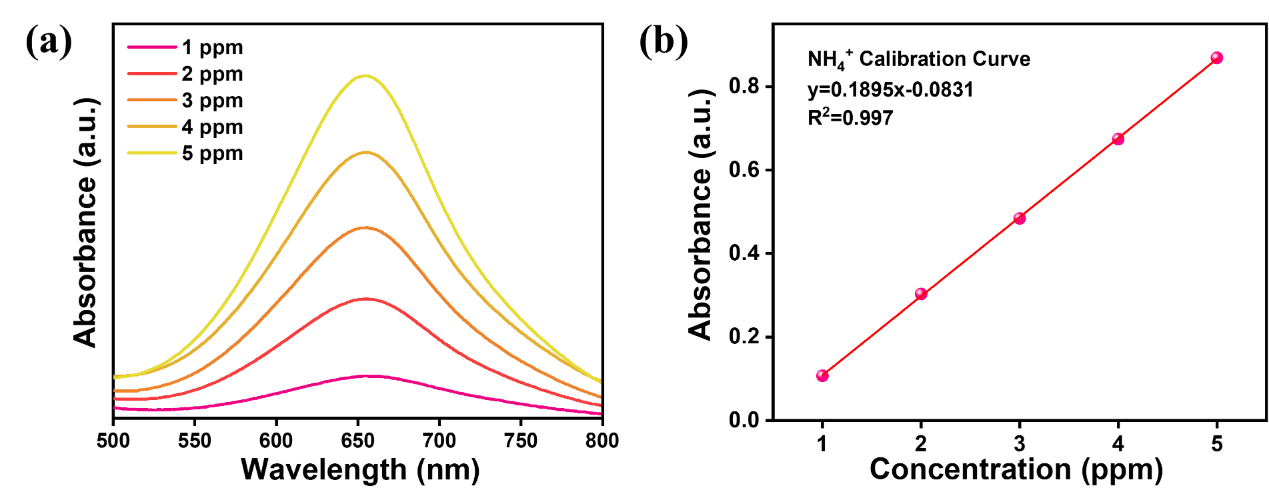


Fig. S40 (a) UV-vis absorption spectra of various NH_4_^+^ concentrations, and (b) Calibration curve used for quantifying NH_4_^+^


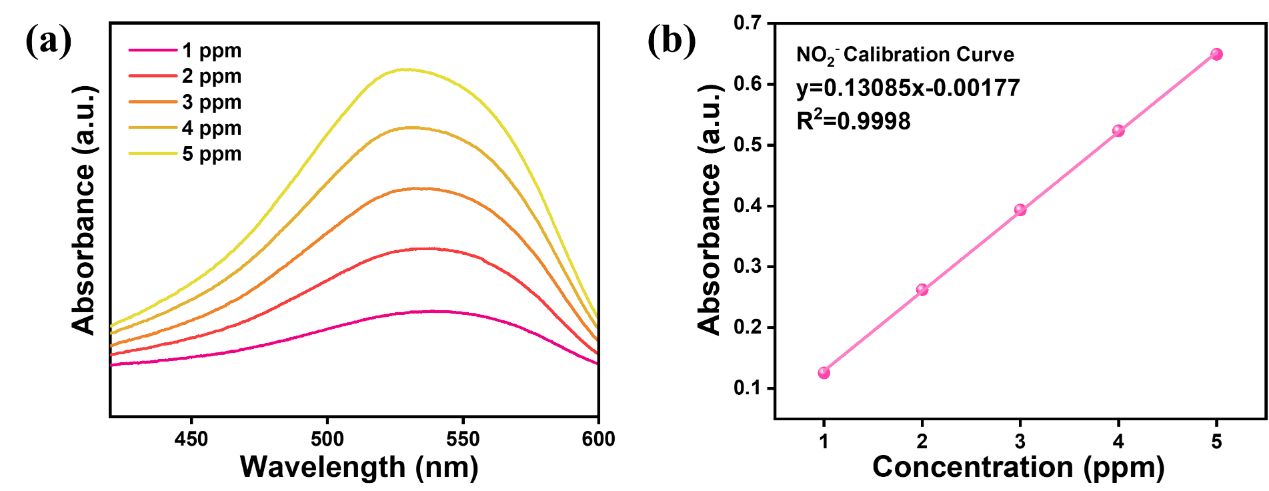


Fig. S41 (a) UV-vis absorption spectra of various NO_2_^–^ concentrations, and (b) Calibration curve used for quantifying NO_2_^–^


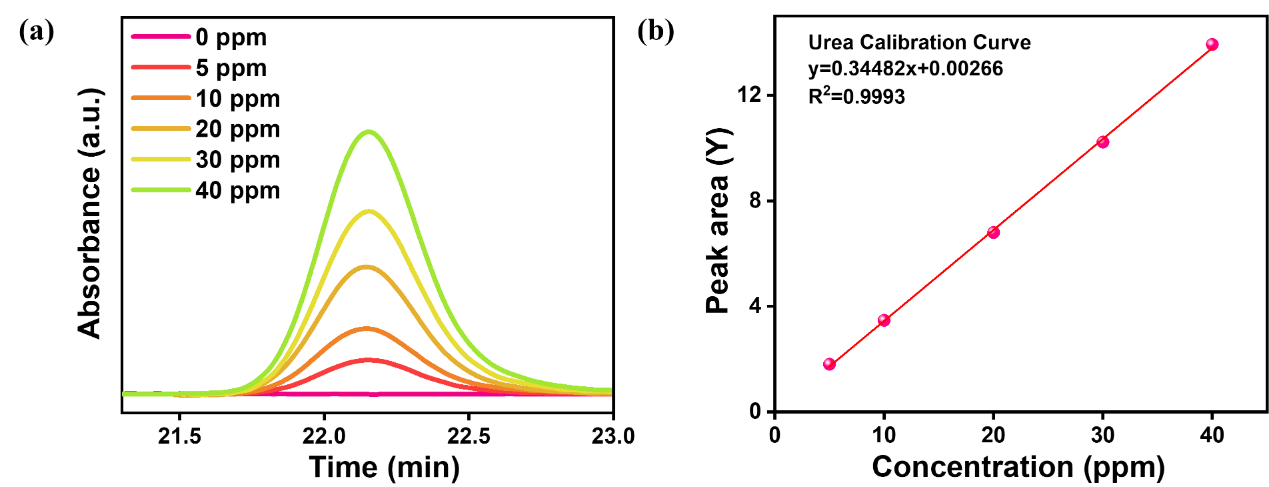


Fig. S42 (a) HPLC absorption spectra of various urea concentrations, and (b) Calibration curve used for quantifying urea


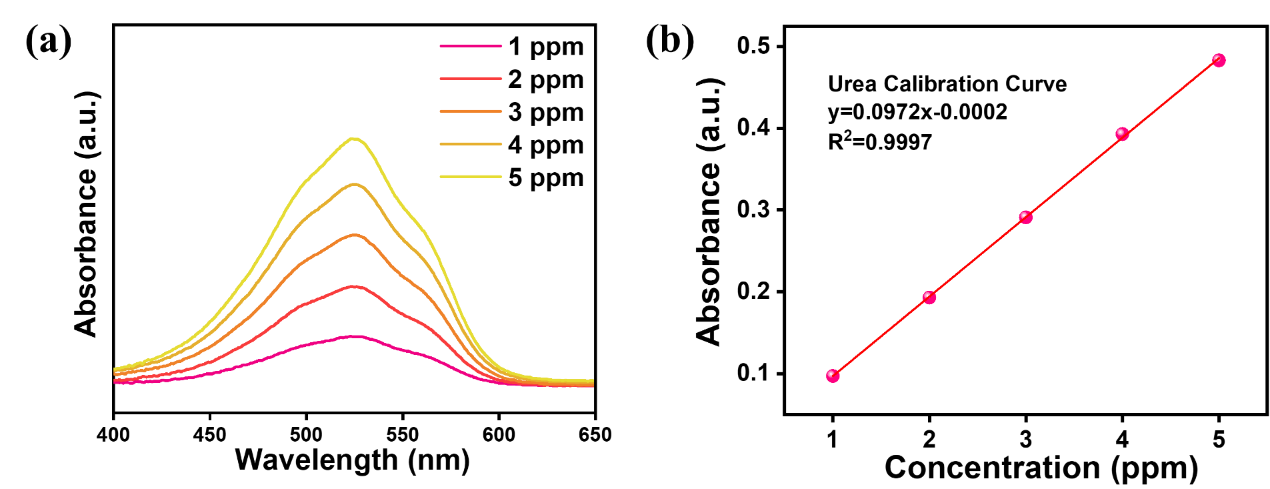


Fig. S43 (a) UV-vis absorption spectra of various urea concentrations, and (b) Calibration curve used for quantifying urea

Table 2 Summary on the FE of electro-catalysis coupling for C–N bond toward urea synthesis

| **Catalyst** | **Reactant** | **E (V vs.**  **RHE)** | **Urea yield**  **(μg mg^-1^ h^-1^)** | **FE** | **Refs.** |
| --- | --- | --- | --- | --- | --- |
| **O-PdZn/C** | **NO_3_^-^+CO_2_** | **-0.4** | **1247.42** | **62.78%** | **This Work** |
| Diatomic  Fe–Ni | NO_3_^-^+CO_2_ | -1.5 | 1213.21 | 17.8% | S2 |
| V_O_-InOOH | NO_3_^-^+CO_2_ | -0.5 | 592.5 | 51% | S3 |
| Zn NBs | NO+CO_2_ | -0.92 | 908.71 | 11.26% | S4 |
| Pd_1_Cu_1_/TiO_2_ | N_2_+CO_2_ | -0.4 | 201.60 | 8.92% | S7 |
| Cu-GS-800 | NO_3_^-^+CO_2_ | -0.9 | 1800.03 | 28% | S8 |
| PdAu alloy | NO_3_^-^+CO_2_ | -0.5 | 204.30 | 15.6% | S9 |
| Cu_97_In_3_-C | NO_3_^-^+CO_2_ | -1.4 | 786.79 | ~5.3% | S10 |
| Fe(a)@C-Fe_3_O_4_/CNTs | NO_3_^-^+CO_2_ | -0.65 | 1341.3 | 16.5% | S11 |
| F-CNT | NO_3_^-^+CO_2_ | -0.65 | 381.6 | 18.0% | S12 |
| Bi/BiVO_4_ | N_2_+CO_2_ | -0.4 | 354.6 | 12.55 | S13 |
| BiFeO_3_/BiVO_4_ | N_2_+CO_2_ | -0.4 | 296.5 | 17.18 | S14 |
| Ni_3_(BO_3_)_2_ | N_2_+CO_2_ | -0.5 | 582.58 | 20.36 | S15 |
| InOOH | N_2_+CO_2_ | -0.4 | 411.41 | 21.97 | S16 |
| CuPc NTs | N_2_+CO_2_ | -0.6 | 143.4 | 12.99 | S17 |
| MoO_x_/C | NO_3_^-^+CO_2_ | -0.6 | 1431.5 | 27.7 | S18 |
| Co–PMDA-2-  mbIM | N_2_+CO_2_ | -0.5 | 868.2 | 49 | S19 |
| Vo-CeO_2_ | NO_3_^-^+CO_2_ | -1.6 | 943.6 | - | S20 |
| Diatomic Zn-Mn | N_2_+CO_2_ | -0.3 | 240.0 | 63.5 | S21 |
| Diatomic Re-Mn | N_2_+CO_2_ | -0.3 | 71.8 | 52.91 | S22 |
| Bimetallic Cu-In | NO_3_^-^+CO_2_ | -1.4 | 786.21 | ~25.3 | S23 |
| Cu–W bimetallic  oxide | NO_3_^-^+CO_2_ | -0.2 | 98.5 | 70.1 | S24 |
| Sb_x_Bi_1-x_O_y_ clusters | N_2_+CO_2_ | −0.3 | 307.97 | 10.9% | S25 |

**Supplementary References**

1. X. Zhang, X. Zhu, S. Bo, C. Chen, M. Qiu et al., Identifying and tailoring C–N coupling site for efficient urea synthesis over diatomic Fe–Ni catalyst. Nat. Commun. **13**(1), 5337 (2022). <https://doi.org/10.1038/s41467-022-33066-6>
2. C. Lv, L. Zhong, H. Liu, Z. Fang, C. Yan et al., Selective electrocatalytic synthesis of urea with nitrate and carbon dioxide. Nat. Sustain. **4**(10), 868-876 (2021). <https://doi.org/10.1038/s41893-021-00741-3>
3. Y. Huang, R. Yang, C. Wang, N. Meng, Y. Shi et al., Direct electrosynthesis of urea from carbon dioxide and nitric oxide. ACS Energy Lett. **7**(1), 284-291 (2022). <https://doi.org/10.1021/acsenergylett.1c02471>
4. G. Kresse, D. Joubert. From ultrasoft pseudopotentials to the projector augmented-wave method. Physical Review B. **59**(3), 1758-1775 (1999). <https://doi.org/10.1103/PhysRevB.59.1758>
5. J. P. Perdew, K. Burke, M. Ernzerhof. Generalized gradient approximation made simple. Phys. Rev. Lett. **77**(18), 3865-3868 (1996). <https://doi.org/10.1103/PhysRevLett.77.3865>
6. G. Henkelman, B. P. Uberuaga, H. Jónsson. A climbing image nudged elastic band method for finding saddle points and minimum energy paths. The J. of Chem. Phys. **113**(22), 9901-9904 (2000). <https://doi.org/10.1063/1.1329672>
7. C. Chen, X. Zhu, X. Wen, Y. Zhou, L. Zhou et al., Coupling N_2_ and CO_2_ in H_2_O to synthesize urea under ambient conditions. Nat. Chem. **12**(8), 717-724 (2020). <https://doi.org/10.1038/s41557-020-0481-9>
8. J. Leverett, T.-P. Thanh, J. A. Yuwono, P. Kumar, C. Kim et al., Tuning the coordination structure of cu-n-c single atom catalysts for simultaneous electrochemical reduction of CO_2_ and NO_3_^–^ to urea. Adv Energy Mater. **12**(32), 2201500 (2022). <https://doi.org/10.1002/aenm.202201500>
9. H. Wang, Y. Jiang, S. Li, F. Gou, X. Liu et al., Realizing efficient c-n coupling via electrochemical co-reduction of CO_2_ and NO_3_^–^ on AuPd nanoalloy to form urea: Key C–N coupling intermediates. Appl. Catal. B: Environ. **318**, 121819 (2022). [/https://doi.org/10.1016/j.apcatb.2022.121819](https://doi.org/https://doi.org/10.1016/j.apcatb.2022.121819)
10. Y. Liu, X. Tu, X. Wei, D. Wang, X. Zhang et al., C-bound or o-bound surface: Which one boosts electrocatalytic urea synthesis? Angew. Chem. Int. Ed. **62**(19), e202300387 (2023). [https://doi.org/10.1002/anie.202300387](https://doi.org/https://doi.org/10.1002/anie.202300387)
11. J. Geng, S. Ji, M. Jin, C. Zhang, M. Xu et al., Ambient electrosynthesis of urea with nitrate and carbon dioxide over iron-based dual-sites. Angew. Chem. Int. Ed. **62**(6), e202210958 (2023). <https://doi.org/10.1002/anie.202210958>
12. X. Liu, P. V. Kumar, Q. Chen, L. Zhao, F. Ye et al., Carbon nanotubes with fluorine-rich surface as metal-free electrocatalyst for effective synthesis of urea from nitrate and CO_2_. Appl. Catal. B: Environ. **316**, 121618 (2022). [https://doi.org/10.1016/j.apcatb.2022.121618](https://doi.org/https://doi.org/10.1016/j.apcatb.2022.121618)
13. M. Yuan, J. Chen, Y. Bai, Z. Liu, J. Zhang et al., Unveiling electrochemical urea synthesis by co-activation of CO_2_ and N_2_ with mott–schottky heterostructure catalysts. Angew. Chem. Int. Ed. **60**(19), 10910-10918 (2021). <https://doi.org/10.1002/anie.202101275>
14. M. Yuan, J. Chen, Y. Bai, Z. Liu, J. Zhang et al., Electrochemical C–N coupling with perovskite hybrids toward efficient urea synthesis. Chem. Sci. **12**(17), 6048-6058 (2021). <https://doi.org/10.1039/d1sc01467f>
15. M. Yuan, J. Chen, H. Zhang, Q. Li, L. Zhou et al., Host-guest molecular interaction promoted urea electrosynthesis over a precisely designed conductive metal-organic framework. Energy Environ. Sci. **15**(5), 2084-2095 (2022). <https://doi.org/10.1039/d1ee03918k>
16. M. Yuan, H. Zhang, Y. Xu, R. Liu, R. Wang et al., Artificial frustrated Lewis pairs facilitating the electrochemical N_2_ and CO_2_ conversion to urea. Chem. Catal. **2**(2), 309-320 (2022). [https://doi.org/10.1016/j.checat.2021.11.009](https://doi.org/https://doi.org/10.1016/j.checat.2021.11.009)
17. J. Meessen. Urea synthesis. Chem. Ing. Tech. **86**(12), 2180-2189 (2014). [https://doi.org/10.1002/cite.201400064](https://doi.org/https://doi.org/10.1002/cite.201400064)
18. M. Sun, G. Wu, J. Jiang, Y. Yang, A. Du et al., Carbon-anchored molybdenum oxide nanoclusters as efficient catalysts for the electrosynthesis of ammonia and urea. Angew. Chem. Int. Ed. **62**(19), e202301957 (2023). [https://doi.org/10.1002/anie.202301957](https://doi.org/https://doi.org/10.1002/anie.202301957)
19. M. Yuan, J. Chen, Y. Xu, R. Liu, T. Zhao et al., Highly selective electroreduction of N_2_ and CO_2_ to urea over artificial frustrated Lewis pairs. Energy Environ. Sci. **14**(12), 6605-6615 (2021). <https://doi.org/10.1039/d1ee02485j>
20. X. Wei, X. Wen, Y. Liu, C. Chen, C. Xie et al., Oxygen vacancy-mediated selective C–N coupling toward electrocatalytic urea synthesis. J. Am. Chem. Soc. **144**(26), 11530-11535 (2022). <https://doi.org/10.1021/jacs.2c03452>
21. X. Zhang, X. Zhu, S. Bo, C. Chen, K. Cheng et al., Electrocatalytic urea synthesis with 63.5 % faradaic efficiency and 100 % N-selectivity via one-step C−N coupling. Angew. Chem. Int. Ed. **62**(33), e202305447 (2023). [https://doi.org/10.1002/anie.202305447](https://doi.org/https://doi.org/10.1002/anie.202305447)
22. X. Zhang, X. Zhu, S. Bo, C. Chen, Q. Zhai et al., Selective nitrogen fixation via janus C−N coupling in co-electrolysis. Chem **10**(5), 1516-1527 (2024). <https://doi.org/https://doi.org/10.1016/j.chempr.2024.01.025>
23. Y. Liu, X. Tu, X. Wei, D. Wang, X. Zhang et al., C-bound or o-bound surface: Which one boosts electrocatalytic urea synthesis? Angew. Chem. Int. Ed. **62**(19) e202300387 (2023). <https://doi.org/10.1002/anie.202300387>
24. Y. Zhao, Y. Ding, W. Li, C. Liu, Y. Li et al., Efficient urea electrosynthesis from carbon dioxide and nitrate via alternating Cu–W bimetallic C–N coupling sites. Nat. Commun. **14**(1), 4491 (2023). <https://doi.org/10.1038/s41467-023-40273-2>
25. X. Chen, S. Lv, J. Kang, Z. Wang, T. Guo et al., Efficient C−N coupling in the direct synthesis of urea from CO_2_ and N_2_ by amorphous sbxbi1-xoy clusters. PNAS **120**(39), e2306841120 (2023). <https://doi.org/10.1073/pnas.2306841120>
